# Supplementary material for: Genome ARTIST: a robust, high-accuracy aligner tool for mapping transposon insertions and self-insertions
Source: Mob DNA. 2016 Feb 5;7:3. doi: 10.1186/s13100-016-0061-0 (PMC4744444; doi:10.1186/s13100-016-0061-0)
Supplement: Additional file 1: — The Genome ARTIST user manual. The manual contains detailed descriptions of data feeding, running parameters, visual outputs and saving options, required for optimally harnessing Genome ARTIST performances. (PDF 702 kb) [file 13100_2016_61_MOESM1_ESM.pdf]

User manual

Genome ARTIST

Version 1.19

Table of Contents

1. Introduction....3
1.1 What is Genome ARTIST....3
1.2 Reporting Bugs....4
2. Installing and Running....4
2.1 Requirements....4
2.2 Installation....6
2.3 Running....6
3. Loading data....6
3.1. Acquiring genomes....6
3.2. Ready-for-use genome files....8
3.3. Transposon files....10
4. Launching a query....12
5. Reading the results....14
5.1 Query Info....15
5.2 Best result....16
5.3 Results table....16
5.4 Result panel (“Best result” or “Result candidate”)....16
5.5 Gene map....18
6. Saving and loading....19
7. Advanced Settings....19
7.1 The search algorithm....19
7.2 Parameters....20
8. License Terms....21
9. Authors of the User manual....21

1. Introduction

1.1 What is Genome ARTIST

Genome ARTIST (**AR**tificial **T**ransposon **I**nsertion **S**ite **T**racker) is a new bioinformatics tool (www.genomeartist.ro) originally developed in order to allow a rapid detection of insertional mutations generated in the genome of *Drosophila melanogaster* by means of artificial *P* element derivatives. Aside from the large gene disruption projects (*FlyBase*, www.flybase.org), many fly laboratories run small scale transposon mutagenesis screenings. Basically, mobilization with a transposase source of artificial molecular constructs (derived from a natural *P* mobile element or from other transposons) induces insertional mutations in the germline. Many different mutant strains are derived from affected parents using classical genetic crosses and, in the end, their putative useful mutations are analyzed by inverse PCR (iPCR) and sequencing. The sequencing product is a mixture of information, where part of it pertains to the fruit fly canonical genome and the rest of it belongs to a specific artificial element. The most critical aspect of sequence analysis is to detect the exact border between the genomic and transposon DNA, equivalent with identification of the insertion site at the nucleotide level. Sequencing products are not always perfect and a few artifact bases mismatches may impair a fluent insertion mapping. Most commonly, the sequences of interest are aligned on-line with BLAST (http://blast.ncbi.nlm.nih.gov) or BLAT (http://www.genome.ucsc.edu) against *D. melanogaster* official genome, without considering either natural or artificial P transposons. Alternatively, dedicated software like *iMapper* (http://www.sanger.ac.uk/cgi-bin/teams/team113/imapper.cgi) are employed, but some limitations regarding customization and sensitivity are encountered. Often, additional manual sequence annotation is needed in order to finish an accurate insertion mapping and here is when **Genome ARTIST** enters the scene and offers a bit of help. The query sequence is simultaneously compared off-line against both the *D. melanogaster* genome and the specific transposon sequence, partial sequence alignments are matched to each other, relative scores of alignments are calculated and the best mix sequence with the genomic and transposon coordinates is offered to the user. Different colors are used for genomic versus transposon fragments, and an intuitive list of results and details is also depicted. One may easily observe the site of insertion relative to the specific genomic release loaded in **Genome ARTIST**, the gene affected by the transposon insertion, and also the genes located in the close vicinity of the insertion. Special biological conditions occurring during mutagenesis experiments, such as transposon reinsertions into the original mobile element copy, are not usually detected with other searching algorithms, therefore

Genome ARTIST is designed to reveal and to interpret such events.

To some extent, **Genome ARTIST** is an alternative for the classical alignment algorithms and may be exploited for checking the specificity of short sequences as primers or probes. Last but not least, aficionados of different model organisms may use the abilities of **Genome ARTIST** by loading other genomes and/or specific transposons. The performances of **Genome ARTIST** were also successfully tested on various genomes as those of *Pseudomonas aeruginosa*, *Saccharomyces cerevisiae*, *Arabidopsis thaliana*, *Caenorhabditis elegans*, *Drosophila pseudoobscura*, *Ciona intestinalis, Danio rerio*. Because of the sheer size of mammal genomes of *Mus musculus* and *Homo sapiens*, only a bit more than half of such a genome may be loaded in a single **Genome ARTIST** package, thus the user should choose the chromosomes of interest for loading. Additionally, pairwise comparative alignments may be performed among sequences pertaining to various species, allowing the identification of structural orthologous genes.

1.2 Reporting Bugs

So... the inherent bugs... We would appreciate any report concerning such annoying things in order to fix them as soon as possible. Please send any comments at alexandruecovoiu@yahoo.com

2. Installing and Running

2.1 Requirements

a. Hardware

RAM memory

- 512 MB should be available in order to use only the *D. melanogaster* genome
- For any additional genome files loaded, the RAM requirements will increase with about the size of the loaded file + 50% of its size

DISK memory

- 700 MB if you plan to use only the *D. melanogaster* genome
- For any additional genome files loaded, the space requirements will increase with approximately (3 * SIZE_OF_LOADED_GENOME_FILE)

Processor

- The lowest performance processor the program has been tested with is an Intel Atom 1 GHz

Genome ARTIST was designed for a 32-bit architecture but users of a 64-bit OS version may employ the software by installing the package *ia32-libs.* For Ubuntu 12.04, one can install this package by running the command *sudo apt-get install ia32-libs*. Starting with Ubuntu 13.10, *ia32-*

***libs* is not supported any longer and the issue is solved by running the command *sudo apt-get install lib32z1 lib32ncurses5 lib32bz2-1.0* or *sudo apt-get install libstdc++6:i386*.**

b. Software

Operation system

- Linux OS

-**Genome ARTIST** has been tested on Ubuntu 10.04-14.04, Linux Mint 14.1, openSUSE 12.3, CentOS 6.4 and Fedora 19 distributions and similar performances were obtained.

Other dependencies:

-JAVA JRE v1.6 or v1.7 (sometimes called v7) which can be installed from Ubuntu Software Center (OpenJDK Java 7 Runtime).

As an alternative, the user may choose Oracle JAVA JDK 7, which can be installed through a PPA repository available at http://www.webupd8.org/2012/01/install-oracle-java-jdk-7-in-ubuntu-via.htm using:

sudo add-apt-repository ppa:webupd8team/java

sudo apt-get update

sudo apt-get install oracle-java7-installer

For smooth-running performances of **Genome ARTIST** we recommend BioLinux 7 workstation based on Ubuntu 12.04 OS (http://nebc.nerc.ac.uk/tools/bio-linux/bio-linux-7-info) which contains pre-installed packages of *ia32-libs* and OpenJDK Java 6 Runtime. BioLinux 7 is tuned for bioinformatics applications and may be also run live from a DVD or a USB stick, if full installation or dual-boot is to be avoided.

We noticed that some automatic updates of BioLinux 7 impair the graphics of the loading bar which display the alignment progression during query searching. This problem may be circumvented by avoiding updates, by running live BioLinux 7 from a DVD or USB live stick, or by removing OpenJDK from BioLinux 7 and installing Oracle JAVA JDK 7 instead, as described above.

Starting with Ubuntu 13.04, care should be taken about a default setting which opens the executables as text. The problem is solved as it follows: open the folder Genome ARTIST, select File/Preferences/Behaviour then for Executable Text Files select either “Run executable text files when they are opened” or “Ask each time”.

2.2 Installation

The program comes as a .zip archive that must be extracted in a folder easy to be accessed. Aside the extraction, there is no other necessary action to be done.

2.3 Running

To start the program, the script "Genome-ARTIST.sh"(**which should be marked as an executable when imported from an external storage device**) must be run. As an alternative, **Genome ARTIST** may be used as an executable from an ext2/ext3/ext4 formatted external storage device. If the user wants to avoid installation of both an OS and a **Genome ARTIST** package in the computer, a combination of a BioLinux 7 USB live stick and a ext2/ext3/ext4 formatted external storage device containing the **Genome-ARTIST** package is the fastest and simplest solution.

3. Loading data

3.1. Acquiring genomes

On the project's website (www.genomeartist.ro) the user can find bundles containing *D. melanogaster* genome or the genomes of some well know model organisms. These genomes can be extracted and loaded using the „Add folder" feature of **Genome ARTIST**.

However, if the genomes found on the website are not relevant, the user can forge specific **Genome ARTIST** friendly genomes. **Genome ARTIST** supports the loading of genomes of *D. melanogaster* and *D. pseudoobscura* from *FlyBase* and also the conversion of many genomes from *Ensembl* (www.ensembl.org) and from *NCBI* (www.ncbi.nlm.nih.gov).

a. Loading data from *FlyBase*

[1] ftp://ftp.flybase.net/genomes/

- From *FlyBase* FTP genome repository [1] download the .raw files and the .fasta files for the genome of choice

- Place all the downloaded files into a single folder and extract all the archives

- Rename the .raw files (example 2L.raw)

- Rename the annotation fasta file using the following syntax: <chromosome name>_gene.fasta (example 2L_gene.fasta). The term "_gene.fasta" is mandatory

- Load the genome into **Genome ARTIST** using the "Settings > Add Folder" path and press "Ok" to finish the uploading. Completion of the task will take between a few minutes for invertebrates

and a several tens of minutes for vertebrates, depending also on the hardware performances.

b. Loading data from *Ensembl*

[2] http://www.ensembl.org/info/data/ftp/index.html

- From *Ensembl* FTP repository [2] download the following files for a given genome:

  - From “DNA sequence > FASTA” download all the chromosome's sequences from the beginning of the list till the folder dna.toplevel.fa.gz which should not be uploaded.

For example, for *D. melanogaster* all the folders starting with Drosophila_melanogaseter.BDGP.5.73.dna.chromsome.2L.fa.gz and ending with Drosophila_melanogaseter.BDGP.5.73.dna.chromsome.dmel_mitochondrion_genome.fa.gz should be downloaded. For the masked version of the genome, the folders from Drosophila_melanogaseter.BDGP.5.73.dna_rm.chromsome.2L.fa.gz and including Drosophila_melanogaseter.BDGP.5.73.dna_rm.chromsome.dmel_mitochondrion_genome.fa.gz should be downloaded. Enough time should be allowed for the output folder to be completed.

The release number (BDGP.5.73) is used just for the sake of the example as it is constantly changing following re-annotation.

  - From “Annotated sequence -> EMBL” download all the archives

- Place all downloaded files into a single folder

- Extract all archives

- From **Genome ARTIST** install folder, copy the two script files found in ./scripts/Ensembl/ into the folder containing the downloaded files

- Ensure that the two script files are marked as executable (chmod + x)

- Run the “parse_ensemble.sh”

- After the script is executed, a folder named “output” will be generated. This folder contains all the required files for loading the genome into **Genome ARTIST**

- Load the genome into **Genome ARTIST** using the “Settings > Add Folder” option, then press “Ok” to finish uploading

c. Loading data from *NCBI*

[3] ftp://ftp.ncbi.nlm.nih.gov/genomes/

- From *NCBI* FTP repository [3] one should download the following files for a genome of interest:

- If the genome of interest contains only one chromosome (as in bacteria), the user should download the file ending with ".gbk"

- If the genome has more than one chromosome, then, for each of them, the user should download the file ending with extension ".gbk"

- Place all downloaded files into a single folder

- From **Genome ARTIST** install folder, copy the three script files found in ./scripts/NCBI/ into the folder containing the downloaded chromosome files

- Ensure that the three script files are marked as executable (chmod + x)

- Run the "parse_ncbi.sh" file

- After the script is executed, a folder named "output" will be generated. This folder contains all the required files for loading the genome and the annotations into **Genome ARTIST**

- Load the genome of interest into **Genome ARTIST** using the "Settings > Add Folder" option, then press "Ok" to finish uploading

NOTE: the scripts described above work only for some of the genomes available in the FTP repository of NCBI.

3.2. Ready-for-use genome files

a. Loading a whole genome

At www.genomeartist.ro you may find **Genome ARTIST** user-friendly, ready-for-use genome files of some experimental models. Just extract the archive of interest in a dedicated folder, then open the Settings Panel in **Genome ARTIST** and, under Genome files, you will find the button „Add folder". You will be prompted for a name and a folder. This name will be used as a suffix for naming the loaded chromosomes (example dmel_2L, dmel_3L, etc.). Select the folder that appeared after extracting the genome archive. After choosing the folder and the name, just press "Ok" and the loading of the genome into the application should start.

Warning: this will take some time depending on the size of the genome and the computer's performances.

After loading a genome you may co-load a different genome if you are interested in comparative sequence analysis, following the same steps as above. For running bacteria or invertebrate genomes 1 GB of RAM is enough, but for individual genomes of vertebrates such as *D. rerio,* at least 3 GB of RAM are required.

b. Loading a chromosome (genome file)

Instead of using “Add folder” option in order to load a whole genome, one may load only one or a few chromosomes (here regarded as genome files) step by step. Loading (even an entire genome) file by file is advantageous since the right association between a sequence and its annotation is manually performed, therefore is not necessary to adjust the names of the downloaded .raw and .fasta files. The names may be kept unchanged, as in the host database.

To load a genome file, access the “Settings” menu and select the “Genome files” tab. Once there, press the “Add file” button and the necessary information will be prompted:

- “Name”: enter the name for the new genome data source
- “Sequence location (.raw)”: press the browse button on the right of the field and select the file containing the raw data (a continuous sequence of nucleotides); only A, a, C, c, G, g, T, t characters are permitted inside the file since any other characters (like the space character, or new line) will cause the file to be processed in a wrong manner
- “Genes location (.fasta)”: press the browse button on the right of the field and select the file containing the genes information corresponding to the .raw file, written in the .fasta format
- The first two fields are required, and the third field is optional

Press the “Ok” button (see Fig. 1) and the program will process your input to create the new genome data source.

Warning: the time interval necessary for creating the new data source depends on the computer performance.

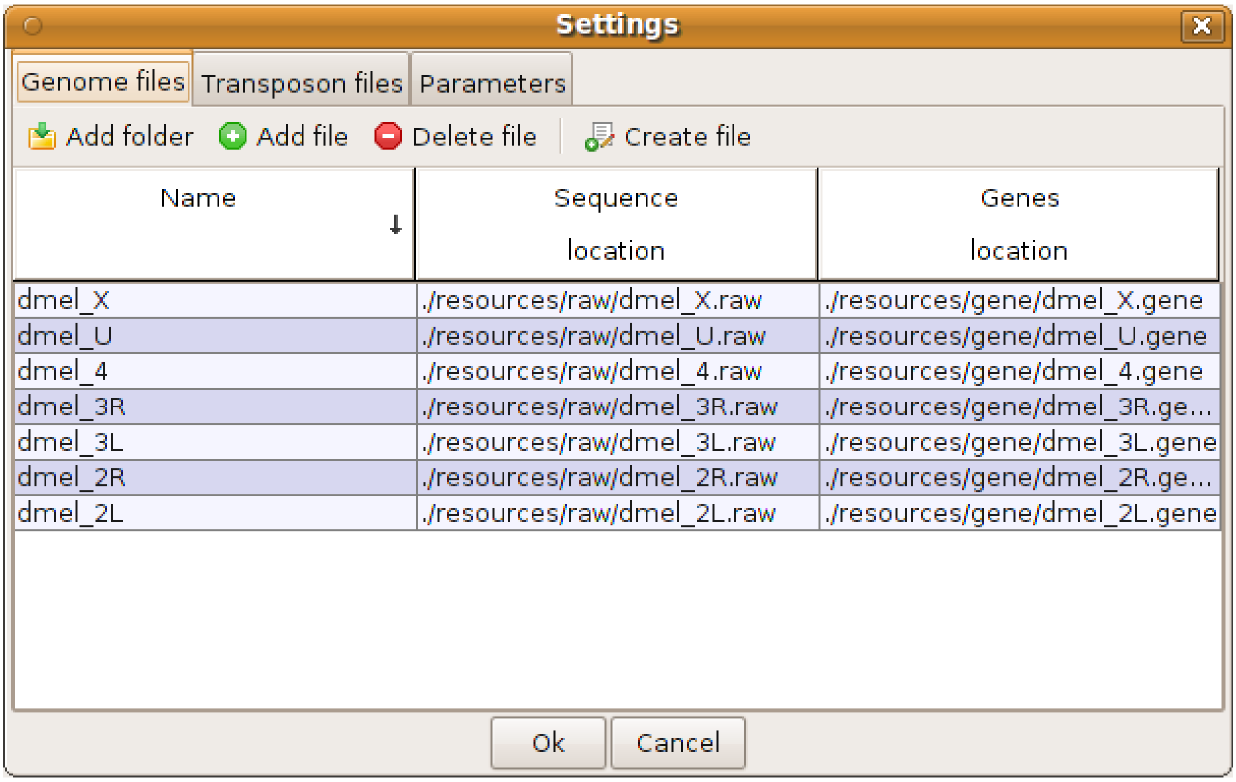

Fig. 1. Pressing “Ok” will load all the genome files (chromosomes) in RAM memory.

c. Creating a new genome data source directly inside the program

To create a new genome data source, access the “Settings” menu, and select the “Genome files” tab. Once there, press the “Create file” button and the necessary information are prompted:

- “Name”: enter the name for the new genome data source
- “Sequence”: enter the nucleotide sequence; only A, a, C, c, G, g, T, t, N, n characters will be kept after processing (it allows for copy/paste from another location that contains spaces, new lines, or any other unnecessary characters)

Press the “Ok” button and the program will process the input to create the new genome data source.

d. Deleting a genome data source

To delete a genome data source, access the “Settings” menu, and select the “Genome files” tab. Once there, select the source to be deleted and press the “Delete file” button. Individual chromosomes selected from a loaded genome can also be deleted.

3.3. Transposon files

a. Loading an existing transposon file

To load a new transposon file, access the “Settings” menu, and select the “Transposon files” tab.

Once there, press the “Add file” button and complete the necessary information:

- “Name”: enter the name for the new transposon data source
- “Sequence location (.raw)”: press the browse button on the right of the field and select the file containing the raw data (a continuous sequence of nucleotides); only A, a, C, c, G, g, T, t characters are permitted inside the file; any other characters (like the space character, or new line) will cause the file to be processed in the wrong manner
- “Genes location (.fasta)”: press the browse button on the right of the field and select the file containing the genes information for the .raw file, written in the .fasta format
- The first two fields are required, and the third field is optional

Press the “Ok” button (Fig. 2) and the program will process the input to create the new transposon data source.

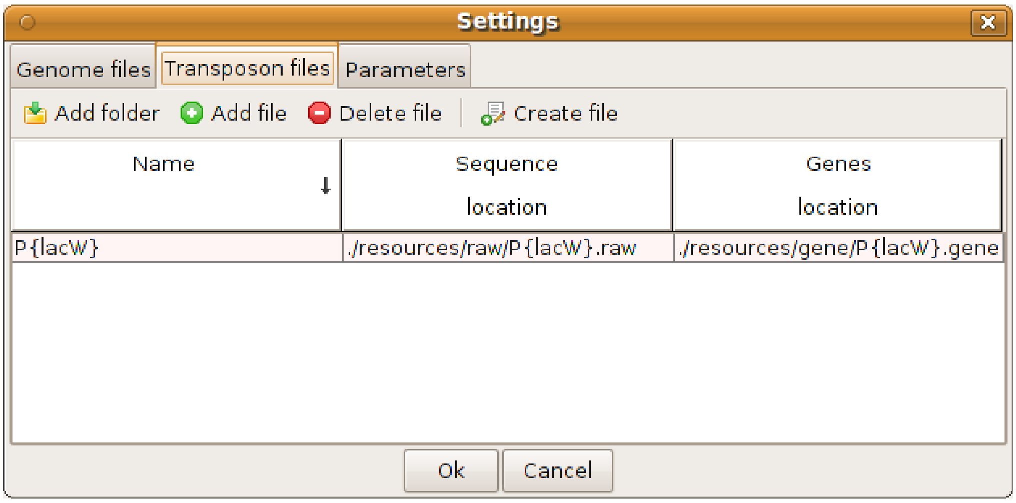

Fig. 2. Loading a transposon file.

b. Creating a new transposon data source directly inside the program

To create a new transposon data source, access the “Settings” menu, and select the “Transposon files” tab. Once there, press the “Create file” button and you will be prompted for the necessary information:

- “Name”: enter the name for the new transposon data source
- “Sequence”: enter the nucleotide sequence; here you can enter any character, but only A, a, C, c, G, g, T, t, N, n characters will be kept after processing (so you can copy/paste from another location that contains spaces, new lines, or any other unnecessary characters)

Press the “Ok” button and the program will process your input to create the new data source

c. Deleting a transposon data source

To delete a transposon data source, you must access the “Settings” menu, and select the “Transposon files” tab. Once there, select the source you want to delete and press the “Delete file” button.

4. Launching a query

To launch a query, press the “New Search” button on the main interface, and complete the necessary information:

- “Query name”: enter the name for the new query
- “Query content”: enter the query to be run

Accepted input:

- A continuous nucleotide sequence
- The query in the *GenBank* or in FASTA format

Starting with version 1.18 of Genome ARTIST, high-throughput alignments are performed when the search window is fed with a list of sequences in FASTA or in *GenBank* format. Similarly, if blocks of nucleotides are separated by at least an empty line in a list of sequences, each block is considered a distinct query, which is individually aligned against the reference sequence and each alignment is reported as a distinct result. Thus, care should be taken in order to avoid accidental empty lines.

We present here a real case of a *P{lacW}* insertion close to *pyd* gene from *D. melanogaster* obtained in our laboratory. In Fig. 3 is depicted the sequence obtained, consecutive to iPCR, with the primer *Sp1* (http://www.fruitfly.org/about/methods/inverse.pcr.html) and in Fig. 4 the reverse complement sequence (simply obtained by checking the dedicated button) is shown. Sometimes, the reverse complement sequence is more intuitive for analysis relative to the reference strand of the genome.

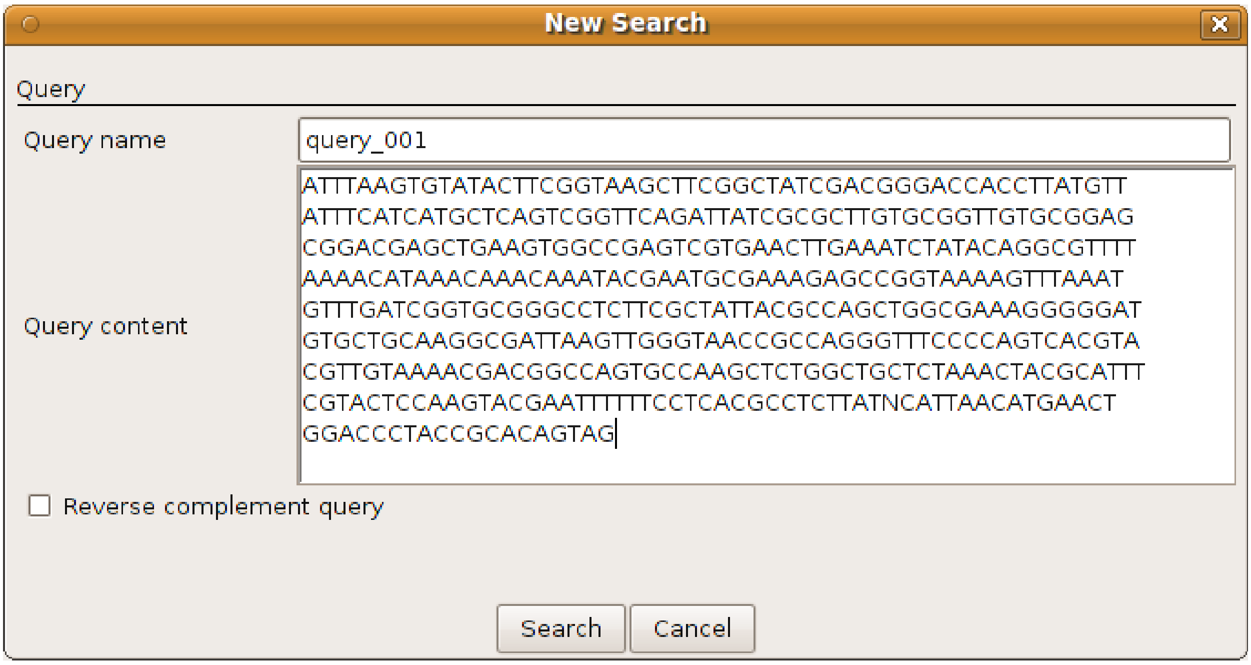

Fig. 3. The original sequence obtained with the primer Sp1.

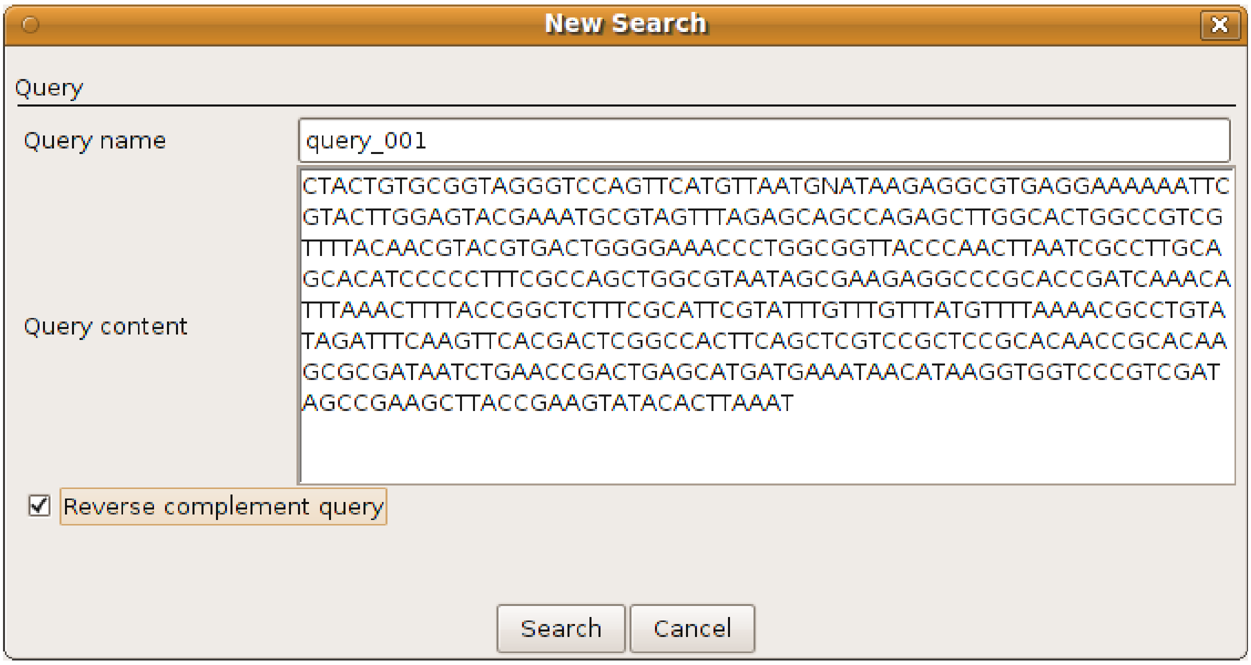

Fig. 4. The reverse complement sequence of pyd associated insertion.

The program will strip away any unnecessary information (like spaces, new lines, *GenBank* or FASTA additional information), and keep only the relevant characters: A, C, G, T and N.

After entering all of the necessary information, press the “Search” button and the prografm will launch the query. While the query is processed, a message will inform that the query is running. After the program computes the results, they will be shown in a new tab, on the main interface.

Warning: to compute the alignment results for a common iPCR-derived sequence query (100-500 nucleotides) it takes between one second and a few tens of seconds, depending on the reference genome size and on the CPU performances (the system must also correspond to the other hardware requirements).

5. Reading the results

The results window has 3 sections as presented in Fig. 5:

- "Query Information"

- "Best result"

- Results table

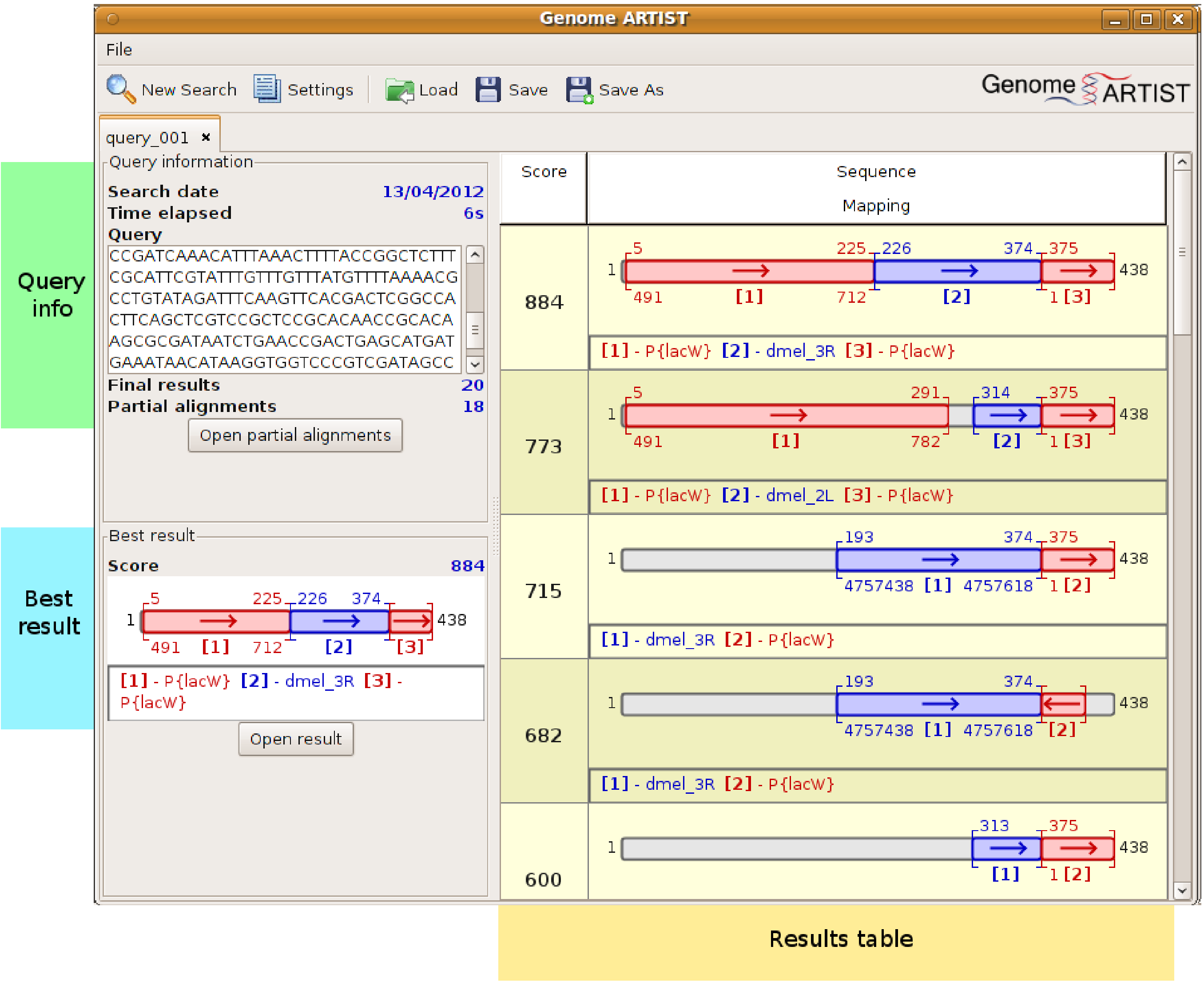

Fig. 5. Details of the results window.

5.1 Query Info

At the top left, summary information about the query can be found. This shows the context in which the query was ran. The summary fields are:

- “Search date” - the date the search was first launched
- “Time elapsed” - the time required for the search to be completed
- “Query” - the sequence that was processed during the search
- “Final results” - the number of results that have been found for this search

NOTE: the maximum number of reported results can be changed from “Settings”.

- “Partial alignments” found - the partial alignments which were used to assemble the final results may be seen by pressing the button “Open partial alignments” (Fig. 6)

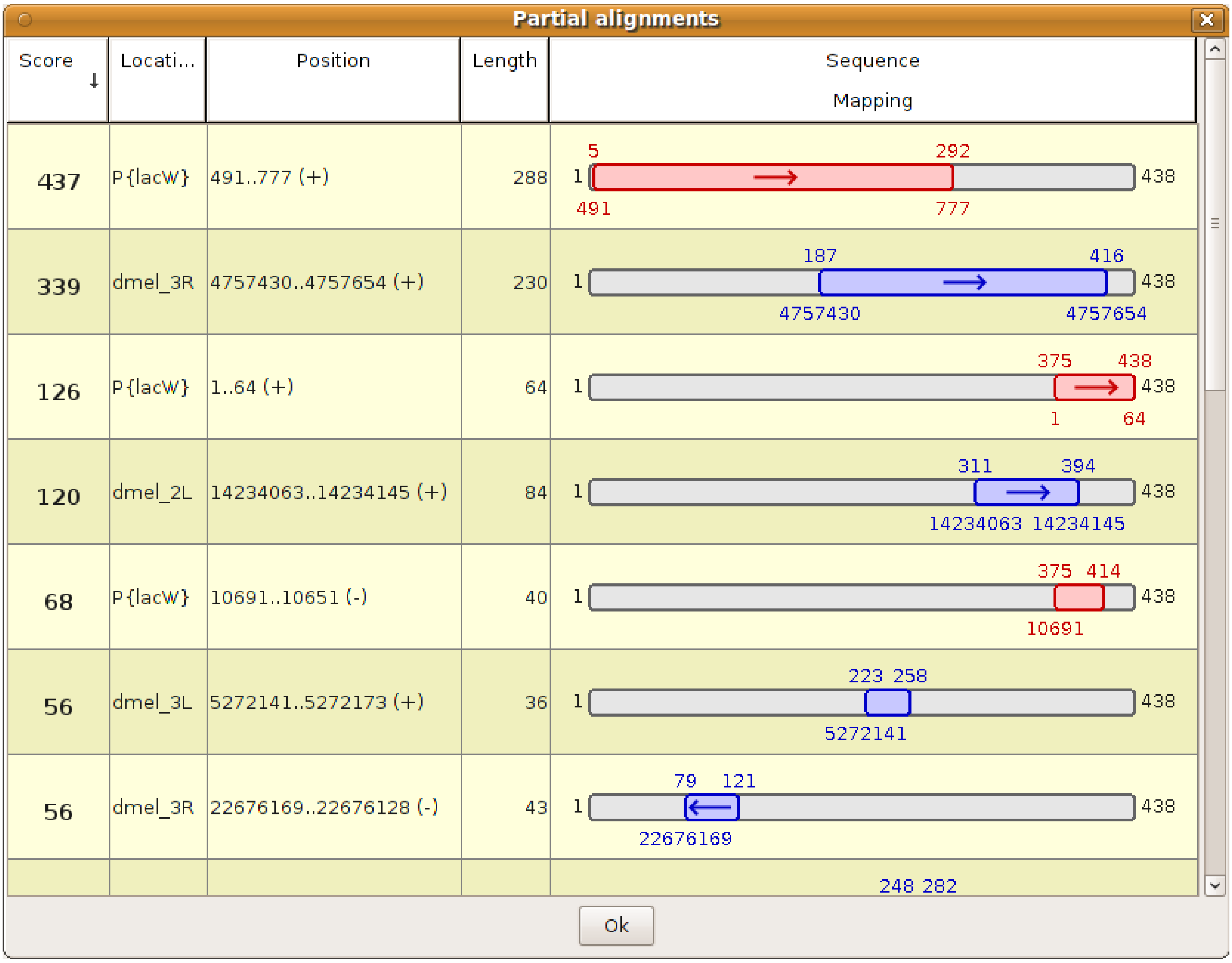

Fig. 6. A list of partial alignments of the query with either genomic (blue) or transposon (magenta) sequences.

5.2 Best result

This panel shows the best proposed results. If, by examining the results, the user finds that another result may be biologically the best, this result can be marked accordingly as "Best result" and it will be shown in the panel.

5.3 Results table

The results table shows the best matches of the given query to the files stored in the database. A result contains "Score" and "Sequence Mapping". The score is proportional to the number of nucleotides that have perfectly matched the query.

The "Sequence Mapping" represents how the genome and transposon sequences matched the query. Genome files and transposon files are differentiated by color (genome sequences are depicted in blue and transposon sequences are shown in magenta). The numbers above the mapping represent the position in the query, while the number below represents the position in the mapped files.

Under each result in "Sequence Mapping" column, there is a legend specifying the files which were mapped and the numbers associated with them are used to visually identify the sequences. The matching strands relative to the reference sequences are also indicated by arrows placed inside the graphical representations of the results. If the arrow is pointing to the right, the sequence matches the forward (or reference) genomic/transposon strand. If the arrow is pointing to the left, then the sequence matches the reverse (reverse-complement) genomic/transposon strand.

This panel shows the minimum amount of information for each query. To find more detailed information about one particular result, double-click on it and a new window, with annotation details, will open.

5.4 Result panel ("Best result" or "Result candidate")

This panel offers several details about a particular result.

It is composed of 3 rows standing for:

- Sequence mapping
- Sequence alignment
- Detailed coordinates and "Annotations"

The sequence mapping section shows the same figure as "Sequence Mapping" in the results table and depicts how specific sub-sequences have matched the given query. The sequence alignment offers details at nucleotide level regarding matches, mismatches and indels.

In Result panel section, the alignments are described along with the nucleotide coordinates pertaining to the specific genome/transposon reference sequence. If more genes are in the area, only one is shown as a green bar.

From this panel, the result can be exported as Image or as PDF or can be printed. Another option is to set any particular result as “Best result”. The result will be shown in the “Best result” panel in the main query window. Using the result panel actually allows the user to locate the exact position of the transposon insertion site (Fig. 7). In the case of *pyd* mutant, the insertion is located in chromosome 3R, at genomic coordinate 4757618 (according to release 5.46 from *FlyBase*) which is placed right near to the nucleotide 1 (the most external one) of *P{lacW}* artificial transposon.

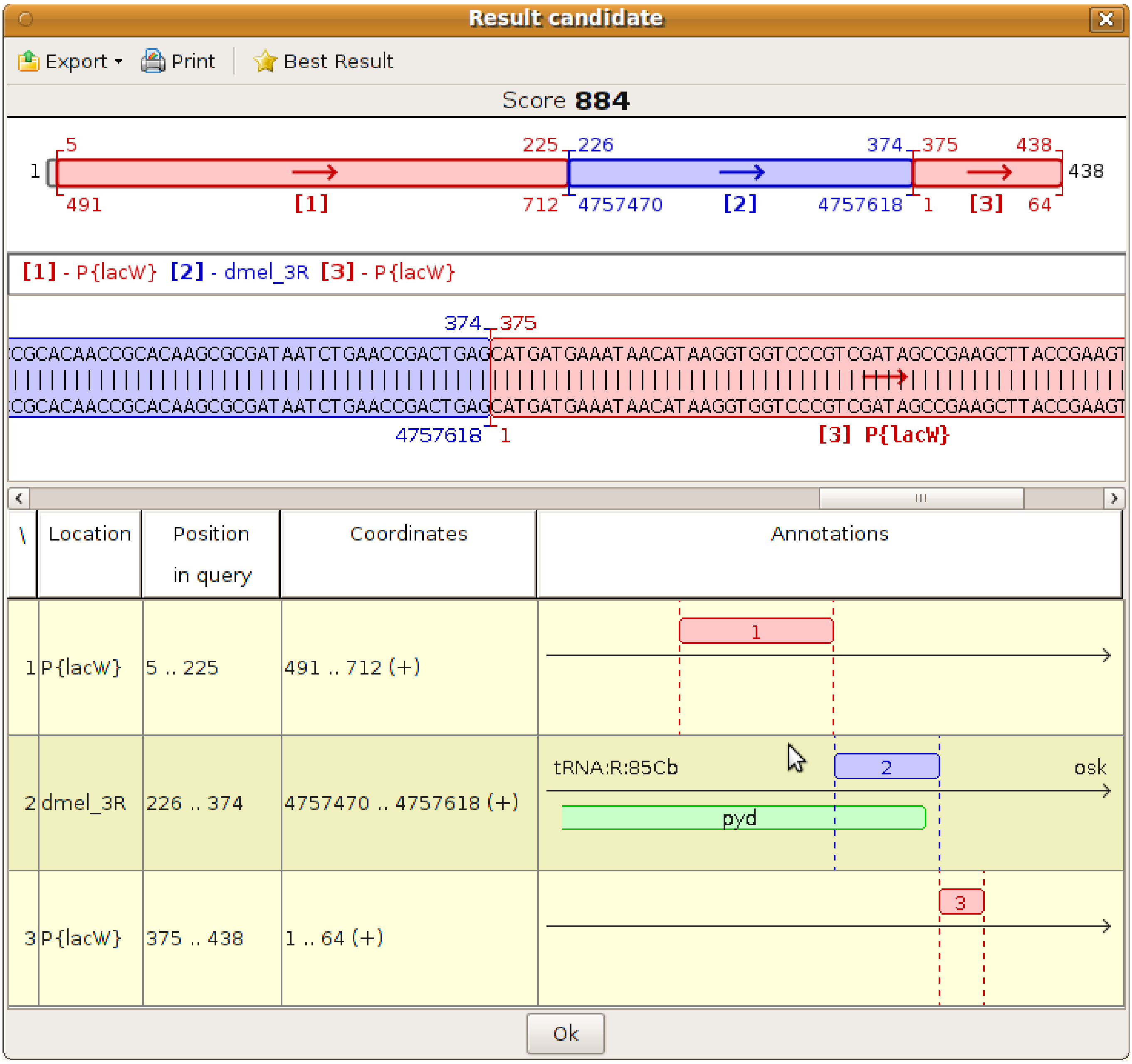

Fig. 7. In the result table panel one may notice that P{lacW} insertion is located close to pyd gene of D. melanogaster, at nucleotide 4757618 (genome release 5.46); nucleotide 1 of P{lacW} artificial transposon (www.flybase.org) coincides with nucleotide 375 of the query.

5.5 Gene map

To obtain more information about the gene hit by the insertion, access “Gene map” window by double-clicking the “Annotation” field. The “Gene map” panel shows the aligned query sequence relative to the local genomic landscape. The arrow shows the relative orientation of the query sequence. At the ends of the arrow, the names of the closest upstream and downstream genes are depicted. Under the graphical view there is a table that briefly describes the genes, their cytological map, their absolute genomic coordinates and location of their sense strands relative to the reference strand of the genome.

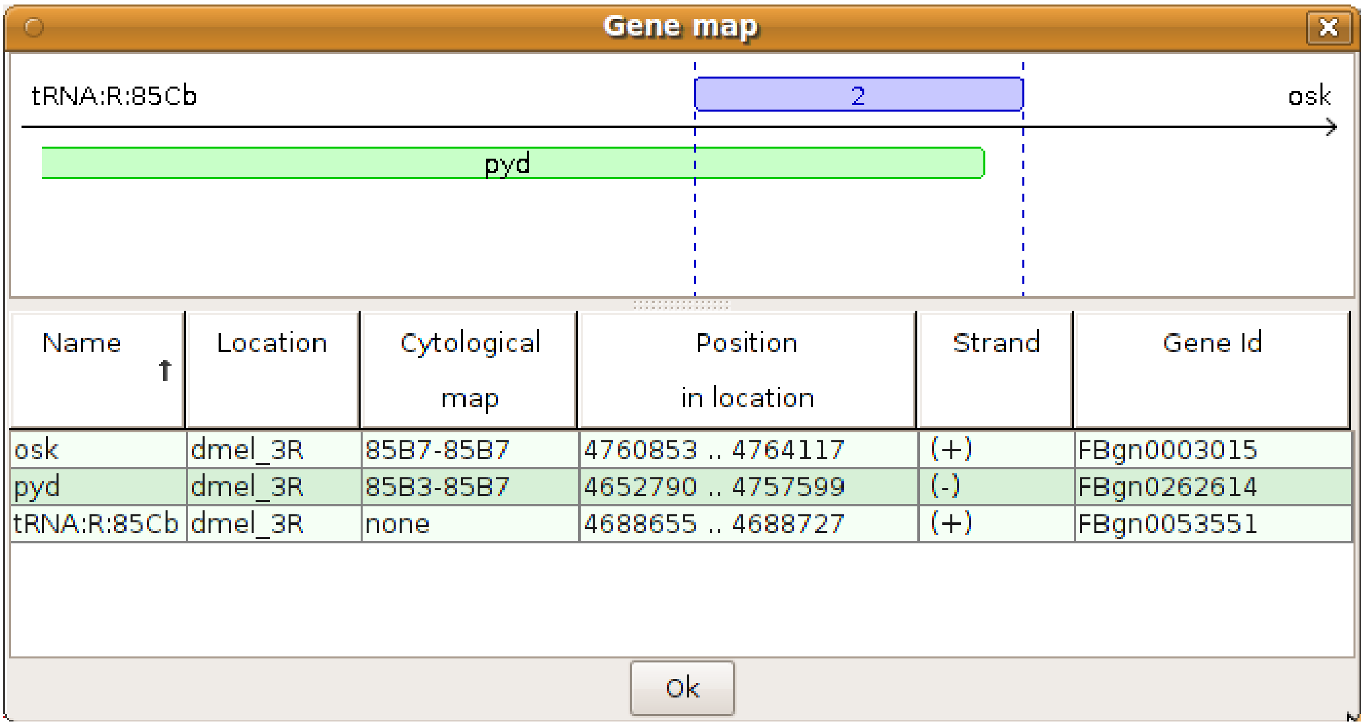

Fig. 8. In the "Gene map" panel it may be noticed that the P{lacW} insertion is located upstream to pyd gene as the sense strand of pyd is on the reverse strand (-) of D. melanogaster genome.

6. Saving and loading

The result that has been found for a given query at specific parameters can be saved by using the "Save" and "Save As" button. Any result is saved in a **Genome ARTIST** format and will have the extension .GA. The files may be stored for a further analysis. The "Load" button works with files having .GA extension and it would load the saved result into the interface.

7. Advanced Settings

The settings for the search algorithm are found in Settings->Parameters.

7.1 The search algorithm

Genome ARTIST uses overlapped intervals of 10 nucleotides (the *k-mers* or decamers), further referred to as *basic intervals*, both for indexing the reference sequences and for spanning the query sequence. When loaded into the built-in database of **Genome ARTIST**, each genome or transposon sequence is hashed and an index of addresses is generated for all of the theoretical 1.048.576 (4^10) distinct decamers. When a comparative search is started, the query sequence is scanned for all its overlapping k-mers then the appropriate index matches (hits) are retrieved. Many initial alignments occur, then the overlapped and/or adjacent basic intervals are fused and *merged continuous intervals*

(*MCIs*) are generated. At this stage, each of the *MCIs* from the query perfectly aligns with a *MCI* from the reference sequence. The *MCIs,* along with some selected basic intervals, are gathered together in a pool which is considered for the next step of the algorithm. Then, an extension step is performed to surpass the alignment imperfections. An initial score of alignment is defined for each of the selected intervals and a window of 4 nucleotides (equivalent to a byte) is used to extend the alignments in the very vicinity of the borders. An implementation of Smith-Waterman algorithm (where a match = 2 and a mismatch = -1) combined with an original formula that penalizes mismatches is used for computing the score of the extension. Any extension stops when the sum of the initial alignment score of an interval and the score of extension drops below zero for each of its borders. Intrinsic to the algorithm, a few mismatches are still incorporated in the resulting *extended intervals* (*EIs*) which are generated this way. When *EIs* are overlapped or adjacent to each other, they are coalesced into *merged extended intervals* (*MEIs*).

After this stage, a list of alignments, also referred to as candidate intervals (*CIs*), is created. The list contains *basic intervals*, *MCIs*, *EIs* and *MEIs* together covering for each nucleotide position in the query. Using again a Smith-Waterman implementation for a rigorous realignment of the *CIs* (a match = 2 and a mismatch = -1), the best scoring alignments are obtained and presented to the user as a list of partial alignments (*PAs*) in the graphical interface of **Genome ARTIST**. Some of the *PAs* pertain to the genome/chromosome and others to the transposon sequence. The most distinctive property of **Genome ARTIST** consists in its capacity to construct alignments referred to as final results (*FRs*) by merging *PAs* of genomic and transposon origin. The *FRs* are shown in an adjustable hierarchical list of alignments and each member may be analyzed in detail by clicking on it. In *FRs* built by merging genome-derived *PAs* with transposon-derived *PAs*, the site of insertion is represented by the genomic nucleotide closest to the first or the last reference nucleotide of a transposon.

7.2 Parameters

The parameters that are found in Parameters tab (Fig. 9.a and Fig. 9.b) are:

"Type of interval expansion" – tells the algorithm how to expand the initial intervals. "Short" means that any mismatches during the expansion process will be badly penalized and "Long" means the algorithm is less severe with the mismatches

"Zero offset" - [Advanced] represents the shift of the score considered neutral for the expansion algorithm. Negative values mean greatly punishing bad alignments

"Match score" - [Expansion] this is a bonus for an exact match in the first position

"Mismatch score" - [Expansion] penalty for a mismatch on the first position

"Length modifier" - [Expansion] the multiplier for the initial score of the expansion phase

"Picking depth" - affects the number of small pieces that are picked as candidates for assembling the final result

"Nucleus size" - represents the minimum size of an interval in the final result

"Number of results" - the number of final results that will be assembled

"Give bonus to insertion candidates" – a bonus is given if the sequence has a transposon flanking a genomic sequence. A transposon end (as an inverted repeat in the case of P mobile element derivatives) must be present at the site of insertion in order to give the bonus

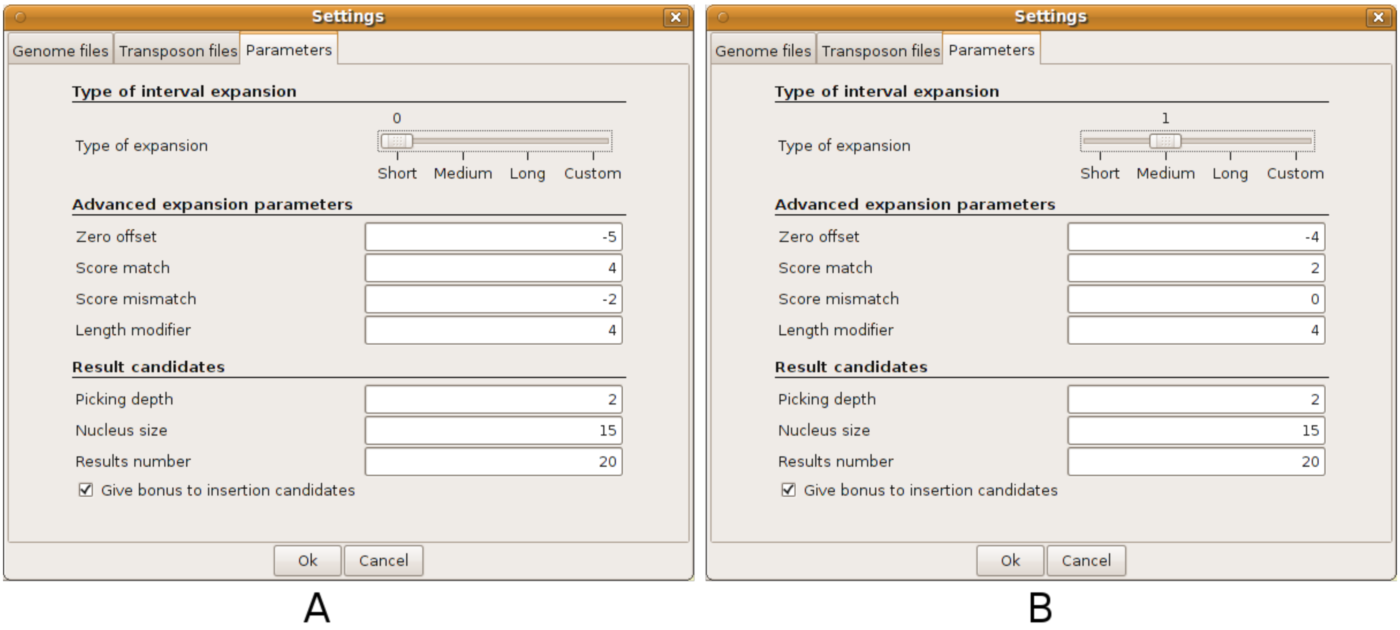

Fig. 9. a) The parameters are set for Short type of expansion; b) The parameters were set for Medium type of expansion, thus the advanced expansion parameters shifted to different values.

8. License Terms

Genome ARTIST is an open source application and is published under GNU General Public License and the source code is freely available at http://www.bioinformatics.org.

9. Authors of the User manual

This manual was co-authored by Ecovoiu Al. Al., and Ghionoiu I. C.
