# Supplementary material for: Genome ARTIST: a robust, high-accuracy aligner tool for mapping transposon insertions and self-insertions
Source: Mob DNA. 2016 Feb 5;7:3. doi: 10.1186/s13100-016-0061-0 (PMC4744444; doi:10.1186/s13100-016-0061-0)
Supplement: Additional file 2: — Detailed mapping strategy and advanced running parameters of Genome ARTIST. The file contains a detailed description of key features of Genome ARTIST, such as the expansion strategy, nucleus definition, alignment parameters and other relevant parameters. (DOC 44 kb) [file 13100_2016_61_MOESM2_ESM.doc]

**Additional file 2**

Genome and transposon reference sequences are uploaded into different databases of Genome ARTIST and hashed to generate an index of addresses for all of the theoretical 1.048.576 (4^10) distinct decamers. Overlapped fragments of 10 nucleotides (decamers), also referred to as *basic intervals* (*BIs*), are used both for indexing the reference sequences and for spanning the query sequence. The decamers are overlapped by 9 nucleotides. When a similarity search is performed, the query sequence is divided in overlapping decamers and then the appropriate matches are retrieved from the index. The overlapped and/or adjacent matching *BIs* are fused, forming *merged continuous intervals* (*MCIs*). In the next step, the *MCIs* and the unmerged *BIs* are considered for alignment extension, which generates *extended intervals* (*EIs*). For this purpose, Genome ARTIST operates with an original scoring strategy relying on the Smith-Waterman (SW) algorithm. This stage surpasses small mutations or sequencing errors which may be encountered when spanning the gaps among *MCIs.* The variables employed for extension may be tweaked in the menu: *Settings*/*Parameters*/*Advanced extension parameters*, or may be preset by selecting among the *Short,* *Medium* or *Long* options, where *short* is the most stringent one and results in the smallest extensions of the *MCIs*.

The alignment expansion is a step by step process which employs overlapped windows of four consecutive nucleotides and starts at one side of a *MCI*/*BI,* which is transformed into a growing *EI.* A *starting score* is considered for each *MCI*/*BI* prior to the first expansion with a window of 4 consecutive nucleotides. This score is obtained by multiplying the number of the matching nucleotides of a *MCI*/*BI* with the value of the parameter *Length modifier*, which is set to 2 by default. Therefore, for a *MCI* of 20 nucleotides, the *starting score* prior to expansion is 40. Different values of *Length modifier* may be employed, depending if the query search is intended for detection of transposon insertion site or for revealing sequences homologies. Higher values of *Length modifier* allow longer extension, while a value of zero results in extension with only a single window of 4 nucleotides at each side of the *MCI.* For each window, an *initial score* is calculated using an original SW implementation in order to obtain the optimal alignment of the four nucleotides. The *initial score* is further used to obtain the *extension score* of the window, which is computed with an exponential formula that incorporates the value of the parameter *Zero offset*. Basically, *Zero offset* is the deviation from a normalized SW score of the nucleotides quartet. When this parameter has negative values (as it is set by default), *Zero offset* acts as an exponential supplemental penalty for mismatching nucleotides occurring in each window. Therefore, the range of alignment extension and the values of the parameter are inversely proportional. The *extension score* also includes either a match bonus or a mismatch penalty score for the nucleotide closest to the growing end of the *MCI*. Practically, when the most stringent preset type of extension is used (*Short*), such a key nucleotide of the quartet is scored +4 for matching and -2 if it corresponds to a mismatch or to a gap. The *extension score* of the first quartet is added to the score of the *MCI* and an intermediary score of the growing *EI* is accounted*.* When extension continues, the second nucleotide of the first window becomes the key first one in the second window, thus each nucleotide is verified and rewarded for matching. Each new window actually overlaps with three nucleotides of the previous one, as it is generated by sliding with one nucleotide outwards relative to the preceding quartet. The *extension score* calculated for each new quartet is added to the last intermediary score of the *EI.* Depending on the matching degree of each new window (0 to 4 perfect nucleotide matches), the intermediary score of the growing *EI* may alternately increase or decrease. When a new added quartet determines the score of the *EI* to drop below zero at the respective growing side, the expansion halts but includes the previous window into the alignment. The same expansion strategy is then employed for the other side of the *EI,* until the score drops again below zero. As a consequence of this strategy, an *EI* alignment may incorporate only relatively few mismatches or small gaps when expansion is performed on *Short* type, as compared when using *Long* type, where milder penalties are involved. The *starting*, *initial*, *expansion* and *intermediary* scores are rather technical terms and are not reported, as they just quantify inter-steps of the expansion stage. However, a basic understanding of how the parameters under *Type of extension* affect the final results may be helpful for the user.

The *EIs* which are overlapped or adjacent to each other are further coalesced into *merged extended intervals* (*MEIs*). A list containing the un-extended *BIs*, un-extended *MCIs*, unmerged *EIs* and *MEIs*, which globally aim to cover each nucleotide position in the query is generated and its members are considered as *candidate intervals* (*CIs*). A second SW implementation is employed for the rigorous alignment of each *CI* over its entire length, using a score scheme where a match is scored as +2 and a mismatch/gap is -1. The optimal alignments addressing the genome and transposon *CIs* are reported to the user as *Partial alignments* (*PAs*), in a list where they may be ordered by SW scores, nucleotide coordinates or length. The main details of each *PA* may be analyzed in the graphical interface of Genome ARTIST. Less stringent values for *Nucleus size* and *Picking depth* parameters, described below, result in more *PAs* selected in the list. *PAs* representing genomic sequences are depicted as blue rectangles and those standing for transposon sequences are depicted as red rectangles. Inside of each *PA* there is an arrow which points to the right when the *PA* refers to reference strand and points to the left when the *PA* corresponds to a reverse strand. In other words, the arrows point to the increasing direction of the nucleotides coordinates.

The final step of the alignment algorithm consists in assembling different *PAs* into *final results* (*FRs*), aiming to cover the entire query sequence. For this purpose, genomic *PAs* (*GPAs*) may be coalesced in order to cover longer genomic intervals. Transposon *PAs* (*TPAs*) may also be merged to obtain longer stretches of transposon alignments. Nevertheless, the most distinctive characteristic of Genome ARTIST is the ability to merge *GPAs* with *TPAs*, in order to construct *FRs* which cover the maximum possible span of the query sequence recovered from an insertional mutant. In order to combine *PAs*, the algorithm relies on parameters such as *Nucleus size*, *Picking depth* and *insertion bonus*.

Each *PA* contains a *nucleus*, which is the longest alignment stretch with high matching density. The nucleus may be either a perfect match of at least 10 nucleotides or an alignment fragment defined by the two outermost stretches of 10 perfectly matching nucleotides of a *PA*. The minimal theoretical value of 10 nucleotides actually represents the length of a *BI*, which is the core searching block employed by Genome ARTIST. In essence, a *nucleus* is constructed around a perfect match of at least 10 nucleotides and its particular size and structure is an intrinsic product of the SW alignment which generated the respective *PA*. For practical reasons, the minimal length of the *nucleus* is adjustable using the *Nucleus size* parameter, which accepts values ranging from 10 to *l* (where *l* is the length of the perfectly matching query). If, for example, *Nucleus size* is set as 30, any of the following alignment structures is a *nucleus*: a perfect *PA* of at least 30 nucleotide pairs; a perfect alignment fragment of at least 30 nucleotide pairs pertaining to a *PA;* an imperfect alignment fragment of minimum 30 nucleotide pairs having gaps/mismatches encompassed by stretches of at least 10 perfectly matching nucleotides. By definition, regardless of its length, each *PA* contains only one *nucleus* and high values for *Nucleus size* reduce the number of *PAs* considered for assembling. If the value of *Nucleus size* is accidentally set below 10, Genome ARTIST still considers 10 (which is the minimal functional value) as the value of *Nucleus size* parameter. When the parameter value is set higher than *l*, Genome ARTIST would report no *PAs* in the list, even if the query sequence of length *l* is *a priori* known to perfectly match a reference sequence from the database*.* In the ideal case, the alignment of a perfectly matching query sequence of length *l* is by itself a single *nucleus* if the parameter *Nucleus size* is set to any value lower or equal to *l*.

The value of the *Picking depth* parameter also determines how many *PAs* would be considered for the assembling step. Each *PA* covers a number of nucleotide matches relative to a query fragment, but some *PAs* are partially redundant when containing common matches*.* When *Picking depth* = +1, each nucleotide position from the query should be ideally covered at least once by matching with a corresponding nucleotide from a *PA*. If the query perfectly matches a stretch of a reference sequence strand, then the coverage is inherently accomplished by the best scoring *PA*. Therefore, at *Picking depth* = +1, the respective *PA* is the only one selected in the partial results list for the respective reference strand and is further used for assembling. Nevertheless, as perfectly matching queries seldom occur, some imperfect *PAs* are generated in the attempt to find partial similarities of a real query with each of the available genomic or transposon reference strands. As a result, for each reference strand, the virtual coverage of the query is fulfilled by a minimal group of *PAs* rather than by the best *PA* present in the list. As an example, if the query coverage could be realized with a set of 3 short *PAs,* but also by a set of 2 longer alternative *PAs,* the second set is selected as it is the minimal one and contains *PAs* with better SW scores. If *Picking depth* = +2, each nucleotide position from the query tends to be covered twice, therefore the set of 3 shorter *PAs* is additionally selected in the list of partial results. Setting high values of *Picking depth* allows for alternative, shorter and less similar *PAs* to be used for assembling. As it is a flexible working option, it may though conduct to false positive results when transposon insertion mapping is on focus. However, it becomes more useful if distant homologies are suspected, as when comparing among interspecific sequences.

The *nuclei* of *PAs* are essential items considered in the assembling process of the *FRs*. If two different *GPAs* are separated by maximum 200 nucleotides on the genomic reference sequence, they are eligible to be merged into the same *FR*. Hence, only indels shorter than 200 nucleotides may be detected with Genome ARTIST. This restriction is not applicable for *TPAs* because Genome ARTIST was also designed to identify self-insertions of the mutagenic transposons. As a result, the distance between the nuclei of *TPAs* may easily exceed 200 nucleotides over the reference sequence of a transposon.

Consequent to the extension step, each nucleus of long *PAs* may be encompassed by lateral stretches of imperfect alignments (*extension tails*), which often overlap to each other*.* In order to offer the best score for a *FR*, *intersection points* between its constitutive *PAs* are calculated and *extension tails* are preferentially adjusted around such borders. Since the *PAs* may thereby undergo apparent modifications, each assembled *PA* is realigned with SW algorithm, a procedure which sometimes slightly modifies the length of the *nuclei.* This is an important aspect, since the basic score of a *FR* is equal to the number of the nucleotide matches contained by the *nuclei* of the assembled *PAs.* If a *GPA* overlaps a *TPA* in a query sequence recovered from an insertional mutant, Genome ARTIST analyses the two *nuclei* and allows an overlapping of maximum 40% between them, but the common sequence is always ascribed to the *TPA*. Hence, the terminal inverted repeat of the integrative element is conserved and it may be graphically presented even when it happens to share nucleotides with the genomic sequence. If the overlapping is not occurring between two nuclei (but it is either present between the *extension tails,* or between *an extension tail* on one side and a *nucleus* on the other), the common alignment is given to the *TPA* (with the limitation that it won’t overrun the nucleus of the *GPA*). If the two *PAs* are of the same type, Genome ARTIST calculates an intersection point as to maximize the score of the alignment solution. This merging strategy allows an accurate mapping of the transposon insertion, even when small scale mutations or sequencing artifacts are present next to the junction border.
