# Supplementary material for: Genome ARTIST: a robust, high-accuracy aligner tool for mapping transposon insertions and self-insertions
Source: Mob DNA. 2016 Feb 5;7:3. doi: 10.1186/s13100-016-0061-0 (PMC4744444; doi:10.1186/s13100-016-0061-0)
Supplement: Additional file 3: — The accession numbers of the real and virtual query sequences. The database accession numbers of the query sequences that were used for testing the mapping performances of Genome ARTIST and iMapper are specified. (DOC 85 kb) [file 13100_2016_61_MOESM3_ESM.doc]

**Additional file 3**

***P{lacW}* reinsertions**:

GenBank:KF361504.2

GenBank:KF361502.2

GenBank:KF361500.2

GenBank:HQ880658.2

GenBank:KF361505.1

GenBank:KF361503.1

GenBank:KF361501.1

GenBank:FJ603693.2

GenBank:HQ695001.1

GenBank:GU814269.1

GenBank:GU814268.1

GenBank:FJ603694.1

GenBank:FJ603692.1

GenBank:FJ603691.1

GenBank:FJ603690.1

GenBank:HM210955.1

GenBank:HM210954.1

GenBank:HM210953.1

GenBank:HM210952.1

GenBank:HM210951.1

GenBank:HM210950.1

GenBank:HM210949.1

GenBank:HM210948.1

GenBank:HM210947.1

GenBank:HM210946.1

GenBank:HM210945.1

GenBank:HM210944.1

***P{EP}* reinsertion**:

GenBank:GU134146.1

GenBank:GU134145.1

GenBank:GU134144.1

GenBank:GU120633.1

GenBank:GQ401364.1

GenBank:GQ401363.1

GenBank:HM216795.1

GenBank:HM210956.1

***P{lacW}* self-insertions**:

GenBank:KM396323.1

GenBank:KM396322.1

GenBank:KM396321.1

***P{lacW}* insertion in opus natural transposon**:

GenBank:KM593302.2

The set of 102 the raw (without simulated SNPs or deletions) query sequences standing for the virtual *P{lacW}* insertions adjacent to the 5’UTR regions of randomly selected *D. melanogaster* genes. In the list, the first term of the FASTA format descriptor (e.g. FBtr0083388) stands for the FlyBase accession number of the full length 5’UTR of the *parent* gene (the nucleotide sequence is depicted with black capital letters). Adjacent to each 5’UTR sequence we manually placed a sequence containing the terminal 41 nucleotides of the *P{lacW}* artificial transposon reference sequence (the nucleotide sequence is in red lower-case letters). The 5’UTRs are naturally lacking introns.

>FBtr0083388 type=five_prime_untranslated_region; loc=3R:complement(12653726..1265**3827**); name=abd-A-RB; MD5=76d226b07edc8a389bce4902b3847b7a; length=102; parent=FBgn0000014; release=r5.57; species=Dmel;

tgtctcttgccgacgggaccaccttatgttatttcatcatgAATCATTCGAACTCAGAAATCTCCGAAAC

TAAAATAGAACAACAAGTGGACTAAACGAAATTCCGAAGATCCGATCCTCGGCCAGAGAG

CAAAATATGTGCA

>FBtr0082786 type=five_prime_untranslated_region; loc=3R:925**2169**..9252325; name=Act87E-RB; MD5=5845e0367f5478c049801d460d92845f; length=157; parent=FBgn0000046; release=r5.57; species=Dmel;

tgtctcttgccgacgggaccaccttatgttatttcatcatgGCAACACCTACACGTATTTCATAATTTCA

CACTTACACACAAGATTACAATTAAAATCCATACCCAATCCGAATACCGAAAGCCCACTT

CTCACTTCTCCTTCTAAAAACCGCCTCCGTTCTCGTTGTTGTTGCAGTGAAAACAGCCAG

TAGCCAAG

>FBtr0084692 type=five_prime_untranslated_region; loc=3R:2057**9571**..20579762; name=asp-RA; MD5=623362867cd30dad4565ea768a885db1; length=192; parent=FBgn0000140; release=r5.57; species=Dmel;

tgtctcttgccgacgggaccaccttatgttatttcatcatgCGAGTATCGGCGTCGCTTCGTTTCGAATT

TCAGTTCGAATTTGGATTTGTGCGGCGACGCTCTAATTTGTTTAATTTTTGTTCGTTAAT

TGTGTTAATTGATTAGTTAGTCGCTGTGTTAATGGACCACTAAGTTAGCTGCGAGCCCGT

TTCTGTTTAGTTCAAGTTATTTCTGTTTTGGCCATTCCCTGCA

>FBtr0081665 type=five_prime_untranslated_region; loc=3R:complement(2584994..258**5188**); name=bcd-RD; MD5=5a50d94d8d745d6cc8fb948e60554673; length=195; parent=FBgn0000166; release=r5.57; species=Dmel;

tgtctcttgccgacgggaccaccttatgttatttcatcatgCATCCCTAAATAACGGCACTCTGCAGATG

CGAAGCAGTGGATCGCAAAAACGCAAAATGTGGGCGAAATAAGTTCGCGAGCGTCTCGAA

AGTAACCGGTTACTGAAAATACAAGAAAGTTTCCACACTCCTTTGCCATTTTTCCGCGCG

GCGCTTGGAAATTCGTAAAGATAACGCGGCGGAGTGTTTGGGGAAA

>FBtr0087783 type=five_prime_untranslated_region; loc=2R:875**7754**..8757907; name=bic-RB; MD5=3c6282fe5bf694bc6844614e8cf9af72; length=154; parent=FBgn0000181; release=r5.57; species=Dmel;

tgtctcttgccgacgggaccaccttatgttatttcatcatgGCGATTTCTTTTTGACATTTGACTACGAC

TGGGCGCACGTGTTTTCTGGCAAGGCTCTGTGCTAAATTCTAGTAAAATCAAAGTGAGTA

GTGGAAAGTGCGGCATTCGATTGGCGTTTTACTGATGCGACCATTGTGTGTACTCAATTG

AACAG

>FBtr0074728 type=five_prime_untranslated_region; loc=X:1946**0216**..19460374; name=car-RA; MD5=2c31735202d2c2c341d494c9fbf35d26; length=159; parent=FBgn0000257; release=r5.57; species=Dmel;

tgtctcttgccgacgggaccaccttatgttatttcatcatgGCGATTACAGTCGCGTCCCTGATCTTCTG

CAGCCGAAATCCGTGGGCGTTGCACCAACACCACCAACGCACTGCACAAACCGCCCACCA

AGGACACGGTAGCGACCGACCGGAAGCCGCAGAGCATACGAATCAACACTTCTGGCAAGA

GGAAAGGAGC

>FBtr0085727 type=five_prime_untranslated_region; loc=3R:complement(26522691..2652**2860**); name=Pka-C2-RB; MD5=be6d8bfd081d8b9a39b8388126da5c9d; length=170; parent=FBgn0000274; release=r5.57; species=Dmel;

tgtctcttgccgacgggaccaccttatgttatttcatcatgCCGATTATCTGATTAATGAGTAAAATTAC

TGAAATCCAAATTTCTAGTGTATCAAGCGCCGATTACCGAATAGATTTGCCACTCAAAAT

CGTTTCGCTAGTCCAGCTTTGCAACGGAAGGCACTCGCAAAGGCGCCGTAAGATGCGCGA

TTGGCCGGATGTTATGCTGTT

>FBtr0079095 type=five_prime_untranslated_region; loc=2L:55**20260**..5520400; name=cl-RA; MD5=2476780feca91d3669e7b6b78ba5b952; length=141; parent=FBgn0000318; release=r5.57; species=Dmel;

tgtctcttgccgacgggaccaccttatgttatttcatcatgTCAGAAAACCTGATAAATTAGGAGAAGTC

CATTCCCCTCCAGAAGCCAGTTACTCCAAAGTTCGCCATCACGTCGCTTCCAACGCTTTT

CGGTCGCATACTCAGACGCTACTGCGCGGTGAATACTAATCCCAGCAAGATC

>FBtr0071687 type=five_prime_untranslated_region; loc=2R:complement(17506478..1750**6584**); name=clt-RA; MD5=a15b1ca26da731f7dfc5035531f44ee4; length=107; parent=FBgn0000326; release=r5.57; species=Dmel;

tgtctcttgccgacgggaccaccttatgttatttcatcatgCAGCTGATTTCGACGAACAAAGTTCAGTG

TTTCAGATTCAGGTTTTGAGATTGCCGAATAATTGCCGATTAGATTGTTAAGGAAACCCG

TAACGGGCTGATTTCACA

>FBtr0336916 type=five_prime_untranslated_region; loc=2R:complement(3274485..327**4620**); name=cos-RB; MD5=2ba0bdba45497626c5977ca68ba05cf9; length=136; parent=FBgn0000352; release=r5.57; species=Dmel;

tgtctcttgccgacgggaccaccttatgttatttcatcatgGGCCACACTATCACATTGTATCGATTCGG

AGGGAGTTGAATTTTTGCACGTATTTGAAAGCGCAAAGATGCGAAACAAAGAGCAGGATA

TCGAATGAAAGAGCTCCTGCGAGACCAGGACTAGTTGCCTAACCACC

>FBtr0333034 type=five_prime_untranslated_region; loc=3L:complement(14170439..1417**0637**); name=D-RB; MD5=b9ea7370fde07060666f8d99d46144fc; length=199; parent=FBgn0000411; release=r5.57; species=Dmel;

tgtctcttgccgacgggaccaccttatgttatttcatcatgATCAGTCACAAAAAAGCAGTCGATCCGAG

CAGAAGCAAAGCAAGAGCAAAGCAGAAGCAAAAGCAAGACAGTAATTGACATAGAGACAA

AGAAAAAATCTGAGAAATCACAGTTGAACCACAGACATATTGTGTTCTAGAAAGTGAAGT

GTTGATCTTGAAAGAAGCAAAAGTTAAACAAAGTGTTCAAAATAGCTGGA

>FBtr0081260 type=five_prime_untranslated_region; loc=2L:1961**1410**..19611526; name=Lar-RA; MD5=99a2686f8aa22d3884e0f57ec1a3ab4a; length=117; parent=FBgn0000464; release=r5.57; species=Dmel;

tgtctcttgccgacgggaccaccttatgttatttcatcatgAAAGAGAATAAAAAGAGCATTTAAAGTTA

TTAGTAACCATCGTTGTTGTTGAGTTGTTGTTGTTGTTACCAACTGTCGACATACCCTTG

CAACTGCCGGCGAAAACATAGCGAAATA

>FBtr0089320 type=five_prime_untranslated_region; loc=2L:complement(19768930..1976**9067**); name=bsh-RB; MD5=3d6bd023e24cffbd3eefe4cd87c6fcca; length=138; parent=FBgn0000529; release=r5.57; species=Dmel;

tgtctcttgccgacgggaccaccttatgttatttcatcatgCGACCGTCAACGAGCAAACAACATAAGCG

CGCGCGCGGTCCAAGGACACGTATATATCCCGGCTGGCTGGCACATCTCCTGGCTCCGGT

CTCCATCACTGCTCCTCGTGCTCCTCCTGCTCGGCCATCCTCCAGCGCA

>FBtr0080325 type=five_prime_untranslated_region; loc=2L:complement(11829236..1182**9405**); name=esc-RA; MD5=6711a882892f7dbfa91da0f4ca636d56; length=170; parent=FBgn0000588; release=r5.57; species=Dmel;

tgtctcttgccgacgggaccaccttatgttatttcatcatgCTGGGTATTTTACGCGATCCGTGACTGGG

GTCACACCATTGCCGCCAAAAAAAAACATTTTCATTCGCCTCGTTATTTAAACATTTAAA

AAAAGCATTATCGAATAGTTCATTGAAGTCGCGGCCTAATTTGGGTTTCATTTAACCGTA

CAAACGTGCCGCGCGTTCGAA

>FBtr0086243 type=five_prime_untranslated_region; loc=2R:complement(16557856..1655**7996**); name=exu-RB; MD5=7212a5185f117f80867d9e6ffe39346e; length=141; parent=FBgn0000615; release=r5.57; species=Dmel;

tgtctcttgccgacgggaccaccttatgttatttcatcatgGTCACTTCATATCGAAAATCTAGTGAAAG

CGGTTCGCGTTTTGCAAAGGAAATCGGTTCCTTAAAAAAAAAAAATTCTAGTGACTCAGC

TAATTTCGTTATCCGAATTTTCTAGATTGCTTTTCGCAGATCATATAAGAAA

>FBtr0343428 type=five_prime_untranslated_region; loc=2L:942**6784**..9426934; name=Fbp2-RB; MD5=822f188a0998696a99e56327b565a066; length=151; parent=FBgn0000640; release=r5.57; species=Dmel;

tgtctcttgccgacgggaccaccttatgttatttcatcatgATCACATTAAATGCGCTGGACATCTGGAA

TCGGTGCATCTGTGGTGTTATATAAGAGCAGCCACCTACTAGCTTAACATCAGACAGAGT

TAGTCACTCATCACATCACAATCCAAGCCACGTCAACCCCAAATAATTAACCCCAAGCAA

AA

>FBtr0077192 type=five_prime_untranslated_region; loc=X:2121**0768**..21210920; name=fliI-RA; MD5=4e272f326c75bd649b28345d75a1640b; length=153; parent=FBgn0000709; release=r5.57; species=Dmel;

tgtctcttgccgacgggaccaccttatgttatttcatcatgCATGCCGTTTGCTCGGGTGAGCGGTGAAC

GGTCACCACTTAGTCTTCTTTTTTTTAGTGCAAAAAAAAAACAAAATAAAGAAGAAGAAA

CGAAAAACAGGGCCAGCAGCTTGAGCCCAAATATAAAACAAATATAACATTTTATGTGGT

CAAA

>FBtr0346516 type=five_prime_untranslated_region; loc=3L:1550**2698**..15502835; name=gdl-RC; MD5=83a490c77309fc9674607f1309016f00; length=138; parent=FBgn0001099; release=r5.57; species=Dmel;

tgtctcttgccgacgggaccaccttatgttatttcatcatgCAGCCCTGGCGCTCAGCTGTTCGTGCGGC

GAAGAGGCGAAATTGGAGTGCGGTTGCACAAAACATCACTAAAAACACAAATACTTGGGA

ATATTACACAAATTATTGTAAATAAATAGGAAATCACAATTTAGTTAAG

>FBtr0075697 type=five_prime_untranslated_region; loc=3L:complement(14796360..1479**6460**); name=gnu-RA; MD5=a3ac2b86551cabcfb883f3eea95b62cf; length=101; parent=FBgn0001120; release=r5.57; species=Dmel;

tgtctcttgccgacgggaccaccttatgttatttcatcatgAGCAGTTAATTACAGTTAGTTGCATTTTG

CAAATCTTATTGCACGTTTTTTTTTGTCGTCTTTTTTGTGCTCGTGGAAAATATTATTTG

TAAAATTACCGA

>FBtr0077867 type=five_prime_untranslated_region; loc=2L:complement(1987653..198**7758**); name=Got2-RA; MD5=77f235d22e8d00fa76faf903a561d714; length=106; parent=FBgn0001125; release=r5.57; species=Dmel;

tgtctcttgccgacgggaccaccttatgttatttcatcatgTTTGTCTTTAGTATTCTCTTGAGGCCGGC

GGCGATTGGCAACGTTTATTACAGTTTCTAATTGTTATTAACTTAAAGCTTAGTACAACC

ACCGAGTGCATTTAGCA

>FBtr0079147 type=five_prime_untranslated_region; loc=2L:594**3965**..5944109; name=Gpdh-RC; MD5=0b33d1a4736d9460db107c66dc8ee7a8; length=145; parent=FBgn0001128; release=r5.57; species=Dmel;

tgtctcttgccgacgggaccaccttatgttatttcatcatgAGAATCAGTAGTCGCCGCGGTTTCGACGT

GTCAGTTTGCAGTACAATTACAATTGATTAATCGCGCCAAATCGCGGAGCCAAGTAGTAC

TTAGCAAGTAGCAAGAGGAAGCACATCAGCGAGTCCATCGACAACAATAACAAAAT

>FBtr0070460 type=five_prime_untranslated_region; loc=X:complement(2322886..232**3000**); name=gt-RB; MD5=a75b8b80024103f40fa41223c2b4f39f; length=115; parent=FBgn0001150; release=r5.57; species=Dmel;

tgtctcttgccgacgggaccaccttatgttatttcatcatgAATCAGTTTGCGTTCGACATCGTCAGCGT

GAAGATATAGCAACATCAGAGCTAGATCACCAGTCTATATAGCGTCAATCAGTTGGATTA

AACCCAGAGACCATACACCGAACACC

>FBtr0076811 type=five_prime_untranslated_region; loc=3L:775**3553**..7753726; name=Hn-RA; MD5=8c83b608a3bb80a147dec94daa6467e5; length=174; parent=FBgn0001208; release=r5.57; species=Dmel;

tgtctcttgccgacgggaccaccttatgttatttcatcatgGGTTTCAGTTCGGATTGATTTTCGTTTGG

GCTCAGCTATGGGGCCTATCGAAAAGTCTATTTAAGCGCGCGATTCATAGAGAAATTAAA

TCAGTTTTAATTCGTTTGCTGAACGGATCGTTCGCCATCGGTTTTATTGTACTTATCAGT

GGAGAAACCCGAGAATCTTGTGAAA

>FBtr0075762 type=five_prime_untranslated_region; loc=3L:1396**7553**..13967670; name=Hsc70-1-RA; MD5=d65de241af865171db3087d573fc7b36; length=118; parent=FBgn0001216; release=r5.57; species=Dmel;

tgtctcttgccgacgggaccaccttatgttatttcatcatgCAGTGATTCTAATTTCATTTTCGATTTCT

ATTCCGTTTCGGCTCGCCTTATTCTCGTTCTCGATCTCTCGGCGGGACCTTGGACGTGGT

GGTGGGGTCTAATTAAAGCCACAGCCAAG

>FBtr0076496 type=five_prime_untranslated_region; loc=3L:complement(9370289..937**0475**); name=Hsp26-RA; MD5=2177e478abb3efeceb95ff06d42e3d58; length=187; parent=FBgn0001225; release=r5.57; species=Dmel;

tgtctcttgccgacgggaccaccttatgttatttcatcatgAGCACAGATCGAATTCAAAAATCGAGCAG

TGAACAACTCAAAGCAACTTTGCGCAAAAGCAAAACTTCAAACGAGAAAAAAAAGGATTA

AAAACCTTTGCTTACAAGTCAAACAAGTTCATTCAACTTAACCAAAGAAAAAATATTTCA

ATCTCGCAAAAGGAACATAACCTAAAGGAAACGTAAAA

>FBtr0070419 type=five_prime_untranslated_region; loc=X:complement(2135936..213**6068**); name=kz-RA; MD5=281d0dc69c321aa7a19f3a23461f2695; length=133; parent=FBgn0001330; release=r5.57; species=Dmel;

tgtctcttgccgacgggaccaccttatgttatttcatcatgAACTTTTGTTATCGCCTGCGCTGTTATCG

ATAAGCGTACTTCGCACGTGTGATTTAAATCAATGTTTATATTATTGCCCAAAGTTAATT

CTGTTACTTTATTTTATTAACTATATTGGTAAAGTAAAGTAATC

>FBtr0079179 type=five_prime_untranslated_region; loc=2L:598**6348**..5986494; name=ifc-RA; MD5=e72d8f299ce7c15cf53352cc587630c2; length=147; parent=FBgn0001941; release=r5.57; species=Dmel;

tgtctcttgccgacgggaccaccttatgttatttcatcatgTCAGGTCATATTAATTTTTCAATCCGCCC

AGTTGGATGTTTGGACAGTTTCCTTCCTCGCGCCGGTCGATAATGTTGGCCAACTAACAC

CGACCTGTAGCACGTCACCCGTAATAACCCATAACCGCAACCAGTCCAAGCATCCAAG

>FBtr0080740 type=five_prime_untranslated_region; loc=2L:complement(15425431..1542**5580**); name=wor-RA; MD5=47186840c6a3350bcb3a9fffe5a07995; length=150; parent=FBgn0001983; release=r5.57; species=Dmel;

tgtctcttgccgacgggaccaccttatgttatttcatcatgGTTGCATCATTCGAGCAAGGAACTCCAAG

CGATCGAGACGCGTGTGGCGAAAGCAAATGAGTGAGCAGTGATTTAAGTTCAAGGATCGG

TATTAAATACAAAATCGAAGATCAGTGATCAGTAACCATAAGGAATAATAACATAGCAAC

C

>FBtr0343812 type=five_prime_untranslated_region; loc=2L:1629**4183**..16294350; name=Cyp303a1-RB; MD5=5b23db4a33290e49a10a80bc2cc2511d; length=168; parent=FBgn0001992; release=r5.57; species=Dmel;

tgtctcttgccgacgggaccaccttatgttatttcatcatgACCGAGCGGATTCAGTCGATTTGAGTGAA

CGTCAGGAGCGGGATCGAACTGTGGACGAAGTCACCAGGAAGCAGTGGATAGCTTACCAA

CCCGAAAGTCCAGCTCGCAACTCCAGCCAAGATTGCGTAACTGGTAGAAAGTGAAAGCAA

AGGCAAAGCGAAAAAAAAC

>FBtr0309834 type=five_prime_untranslated_region; loc=2L:complement(19082101..1908**2221**); name=Lim3-RE; MD5=b4472b4328e97ba3b238e718dce3860e; length=121; parent=FBgn0002023; release=r5.57; species=Dmel;

tgtctcttgccgacgggaccaccttatgttatttcatcatgAATCAAATTCATTTTTGATTAAAATAGCT

CAATTAACAGTTGTCTTGACTCGGTTGTGTGCGTGCATAGGCAAAAATATTTTACTGAAA

CGAAATCGAAAATATAAAATTTTAAGAACAAA

>FBtr0111125 type=five_prime_untranslated_region; loc=2L:2281**7527**..22817627; name=lt-RA; MD5=27d8a8a6d647d59cee41d170aced412c; length=101; parent=FBgn0002566; release=r5.57; species=Dmel;

tgtctcttgccgacgggaccaccttatgttatttcatcatgTATTATTGTCTATGGCGCTATTAGTAAAT

GTAAATAATAAAATAATACATCAGCTTGTGTATTTTCGTTGTTTGAAAATCAGAAATTTC

CGTTTGCTTTAA

>FBtr0077306 type=five_prime_untranslated_region; loc=X:2030**1162**..20301280; name=mal-RA; MD5=b73c3d3b0e6c7530921498c92ae2ab5c; length=119; parent=FBgn0002641; release=r5.57; species=Dmel;

tgtctcttgccgacgggaccaccttatgttatttcatcatgAGTTGATGTATACTGTCCGTGGCTTTTTC

CAGCGAAGAAGAGTATGGCGCAACAAATGCAAGCCGGTCGCAAATAAATAAAGCCCGCTT

TGCAGCCAACACGAATAGTTAGTTCCCAAC

>FBtr0331445 type=five_prime_untranslated_region; loc=2L:complement(16261617..1626**1764**); name=twe-RC; MD5=42b98a290ac4d81ffcfaad9cf49b4579; length=148; parent=FBgn0002673; release=r5.57; species=Dmel;

tgtctcttgccgacgggaccaccttatgttatttcatcatgCATCGGTAGAATGGGGAATTGATAAAGGC

GCCAATAAAGTTGACCGCCAAAAGCGAAACAAACAAGTCGACAGAACAAGAAAATCGTCA

AGGATTTGGCAATCGAAATCACTGCGCACGCTGGAACCCAAAATCTGAACTTCCCAATA

>FBtr0084982 type=five_prime_untranslated_region; loc=3R:complement(21831616..2183**1745**); name=E(spl)mbeta-HLH-RA; MD5=cc41971562ed19f21bfb1dd89f5e3f5e; length=130; parent=FBgn0002733; release=r5.57; species=Dmel;

tgtctcttgccgacgggaccaccttatgttatttcatcatgATTCAAAGCTCCGACTTCGTTGCGTGCAC

ACAGAGTCTCCGAGTCCGAATCTAGTGGAACGTTGCGACTGTGGATTACGCGAGTTTACT

TTACCCCCAATAAAAAAAAAACAAAACCAAACTACAACAAA

>FBtr0084954 type=five_prime_untranslated_region; loc=3R:2182**3343**..21823453; name=E(spl)mdelta-HLH-RA; MD5=fcba12c1d9d164d525a7e8e5a8b801c4; length=111; parent=FBgn0002734; release=r5.57; species=Dmel;

tgtctcttgccgacgggaccaccttatgttatttcatcatgGATCATTTCTCAAGTGACTACCGTGCAGT

GCGGAGCGGCAGCAGCGACATCGCAACATCGCAACTTTATTTACAAACAATCACCACACC

AAATCAAAACCCATTATACACA

>FBtr0302550 type=five_prime_untranslated_region; loc=3R:complement(11798535..1179**8635**); name=mor-RB; MD5=13c2123d1a4e88cb6e2f899eb77807b9; length=101; parent=FBgn0002783; release=r5.57; species=Dmel;

tgtctcttgccgacgggaccaccttatgttatttcatcatgATTTAAATACCATCTCTATTTTATCAACG

CAGCGTTTATTGGCTTTTTGGCGTGGAAAAGTGAAAAGATAATTGACAAAAAGTCCACAA

AAGTGCCGCAAC

>FBtr0082158 type=five_prime_untranslated_region; loc=3R:complement(5609968..561**0092**); name=MtnA-RA; MD5=d16066673cfaf5ac642c2d707b007edd; length=125; parent=FBgn0002868; release=r5.57; species=Dmel;

tgtctcttgccgacgggaccaccttatgttatttcatcatgTGCATCAGTTGTGGTCAGCAGCAAAATCA

AGTGAATCATCTCAGTGCAACTAAAGGCCTAAATAGCCCATACCTACCTTTTTTGTAAAC

AAGTGAACAAGTTCGAGGAAATACAACTCAATCAAG

>FBtr0073817 type=five_prime_untranslated_region; loc=X:1361**6279**..13616386; name=mus101-RA; MD5=0b4c16eaeb0f6755633efb31deb0f2e0; length=108; parent=FBgn0002878; release=r5.57; species=Dmel;

tgtctcttgccgacgggaccaccttatgttatttcatcatgCAATAACAAACGGCGCCAAAATAGCGTAC

GCGAAAAAAAGTGCAGTGCGAAAATCACGGCAATTTTGCAGCGCATCACGGAGCAGAGAA

CACACTCGCAACCGCCATC

>FBtr0085534 type=five_prime_untranslated_region; loc=3R:complement(25632349..2563**2469**); name=ncd-RA; MD5=b8f8aef8f914075033e6e693792f7b61; length=121; parent=FBgn0002924; release=r5.57; species=Dmel;

tgtctcttgccgacgggaccaccttatgttatttcatcatgCTGCAATCAAAAATAACGGCGGGAATCGA

CGGCGTCGCTTGTCAACAACGCAAAACTAATTGATAAAATCGGTTGCAAGGAGGCAGACG

TATCTTCTAAGTTAGGCACAACACAGTTGGCG

>FBtr0073516 type=five_prime_untranslated_region; loc=X:1147**4805**..11474956; name=nod-RA; MD5=ba1e6ae2181357cef8dd76071f9e1a92; length=152; parent=FBgn0002948; release=r5.57; species=Dmel;

tgtctcttgccgacgggaccaccttatgttatttcatcatgCAGATCGTTCTTAGTGTTATTACCGCGAC

GGTCACACTGCCAAAATCAATTTGAAATGCAAAGGTCGCTTGGCTTCGTCAAAAAAGTAA

AATAATTACGGTGAATGCAAGCCAATTGTGCATTATTCAAACAACTTCAATTCTTCAATC

TGC

>FBtr0078405 type=five_prime_untranslated_region; loc=3L:complement(21311011..2131**1120**); name=Pc-RA; MD5=6ca34a1556b5fecc0f7b42a39e60af14; length=110; parent=FBgn0003042; release=r5.57; species=Dmel;

tgtctcttgccgacgggaccaccttatgttatttcatcatgCATGAAAATAATCGAGTCGGACGACTATC

GACGTACGCAGAATTGTAAACCAGAAGTTAATTGCAAATAAAACGAATAATAAAACGTTC

CGAGAAGATTATTAATTAAAA

>FBtr0079200 type=five_prime_untranslated_region; loc=2L:612**5132**..6125248; name=Arpc4-RA; MD5=354ced2c57b5892a8c6cfdbd365f5b28; length=117; parent=FBgn0031781; release=r5.57; species=Dmel;

tgtctcttgccgacgggaccaccttatgttatttcatcatgATGGTCACATCTTTTCTGGTTGATAAATT

CCAGGGCCTGTGAATCATAATAAGTTAAGTAAAAAGTGCAGATACAACAAACCGCAGGCA

GCAGACAACAACCCGACACCCAGACACA

>FBtr0079288 type=five_prime_untranslated_region; loc=2L:655**7800**..6557902; name=CoVb-RA; MD5=4d63d275ba24b3f456c83c2c8fe44ddd; length=103; parent=FBgn0031830; release=r5.57; species=Dmel;

tgtctcttgccgacgggaccaccttatgttatttcatcatgTTACTTTACCGCGGTCACACTGATCCATA

GCTGTGTGAAAAATTACGAAAGAATTTTTGGTGTTCACCACTTAAGACCGACCGAAATTT

TCGCAAAACGAACA

>FBtr0303224 type=five_prime_untranslated_region; loc=2L:664**9388**..6649532; name=Tango1-RC; MD5=d0b34f09f2d31e1d237bfcba7c44e607; length=145; parent=FBgn0031842; release=r5.57; species=Dmel;

tgtctcttgccgacgggaccaccttatgttatttcatcatgAGCATTCCTTGGCGCACCCAGTACGGTCA

CACGAATGAAAAGCCAACTCAGCTGAAAGTGAACCCATTTTTATACACAAAAAATTAGTA

GCTGCAGCGAAATAAGTTGATTTCACACGACCACTGCGCCCCGAAAACAATGCAAA

>FBtr0302587 type=five_prime_untranslated_region; loc=2L:740**1335**..7401437; name=CG5171-RD; MD5=1ceebab336648ba0dc4be178e8561742; length=103; parent=FBgn0031907; release=r5.57; species=Dmel;

tgtctcttgccgacgggaccaccttatgttatttcatcatgATTTACCAGAAACTTGGAAACAAGAATCA

AATTGACAGTTTTTCAGTTGGAAAATAAGCCAAATTAAGATTGTGACCCATCAATAAGAG

CAATTCGTTGGAAA

>FBtr0079487 type=five_prime_untranslated_region; loc=2L:778**1465**..7781634; name=CG7164-RA; MD5=cc8f7b0c1a33dff10c87773b2ef3584c; length=170; parent=FBgn0031946; release=r5.57; species=Dmel;

tgtctcttgccgacgggaccaccttatgttatttcatcatgAATAAATCCAAATTATATCAGAACTATTT

TCTAAAATCCTAAAATGAGGCATCGTTCATAAAAAAAAATATATAAAATAAATTCCACAA

TTTTCGACCACTTTGGCAGTCAAAATTCGCAATCTAAATTCGAAATTAGCAAATAACCAT

TTAGCCACAAACAGCGTGACC

>FBtr0079563 type=five_prime_untranslated_region; loc=2L:complement(7860701..786**0839**); name=TwdlE-RA; MD5=aac28791c5ee55b3a2029bab9f04ebca; length=139; parent=FBgn0031957; release=r5.57; species=Dmel;

tgtctcttgccgacgggaccaccttatgttatttcatcatgGTGGCATGCAGTAAGAGGTTCCGCGTCGA

AGCAGCACCACATTGCCAGTAACCAGTAACCAGTAACAGCCAGTAGCACCTAGCCACCTG

GAGCAGTGACAAAACCCGAAACGGATATACCCGAAATAAGCCACCCGAAA

>FBtr0079535 type=five_prime_untranslated_region; loc=2L:802**9462**..8029607; name=baf-RA; MD5=b3cab005f0f9b96b93bfdbfd2bd9655b; length=146; parent=FBgn0031977; release=r5.57; species=Dmel;

tgtctcttgccgacgggaccaccttatgttatttcatcatgCTAGATGTGTTGTTGCATTTTCGAAAATA

TCACTTGCTGCCACACTGGCTGTGCGTCATTTTCAGAAACGCCGCGAATATTTTGCCATT

GAAATCTAGCTAAGCATTGGAATTTCGCAACGTGCAGCAGCAAAGCAAACTACAAAC

>FBtr0079639 type=five_prime_untranslated_region; loc=2L:complement(8196816..819**6918**); name=CG8475-RB; MD5=2365ec5248081e6f8d293d64634ad167; length=103; parent=FBgn0031995; release=r5.57; species=Dmel;

tgtctcttgccgacgggaccaccttatgttatttcatcatgCAATGAAAAGCTGCTCAGCTGTTAGAAGC

GTTTGACTTTTTTTTGACTTTGATACATTTTCTTAAGCGCAATTGCAATATAATTTGCAA

TTGCTGAAGAAACA

>FBtr0079593 type=five_prime_untranslated_region; loc=2L:821**4305**..8214417; name=CG8353-RA; MD5=0689f498562e4f4486d0ea2dc320bd3d; length=113; parent=FBgn0032002; release=r5.57; species=Dmel;

tgtctcttgccgacgggaccaccttatgttatttcatcatgGTCCATTTGCCATCGGACGTTCAAAGGAG

AAACACACGCACTCAACTTGCTAAAAATAATTCAAAATTTAAGAAAATAGGCGTGCCTCT

ATTGACCAAAAGAATTGAACTATA

>FBtr0306392 type=five_prime_untranslated_region; loc=2L:complement(8252852..825**2966**); name=CG8086-RH; MD5=1e82b3feaa2e0cb07e4a2c5f98adc9fa; length=115; parent=FBgn0032010; release=r5.57; species=Dmel;

tgtctcttgccgacgggaccaccttatgttatttcatcatgCTCACACGTGTAAATGTCAGCGAAGGGAG

TTCAGTAAATTTGGTCTTTAACTTTTTTTTTTTACATTGGGGCACAATTTTCAGACTTCG

ATACTACTAAGCCTAAAAACAGCAAG

>FBtr0345779 type=five_prime_untranslated_region; loc=2L:834**6620**..8346804; name=CG7778-RB; MD5=dfd2621005073e205255b2ebb4cc0037; length=185; parent=FBgn0032025; release=r5.57; species=Dmel;

tgtctcttgccgacgggaccaccttatgttatttcatcatgTCAGTTAAATTTCAGAATTCGCAAACTGC

GGTCGCCTTGATATGAAAAATTAATTAAATTTCAATAAACGAGAAGCGCGAAAAACCAAA

CGCAAACGGTCCAATTAAACTAGCATAAATACTATACACTAAACAATTATCTAGAAATAT

ATATAGAATATATTTATTTCGAAATAACCAAATACC

>FBtr0079657 type=five_prime_untranslated_region; loc=2L:838**6139**..8386246; name=CG13384-RB; MD5=64e08f12e2e1bafb90508ae73c1a25d7; length=108; parent=FBgn0032036; release=r5.57; species=Dmel;

tgtctcttgccgacgggaccaccttatgttatttcatcatgTTAGTTTGTTTCGTGCAGCCGGAGAGGGC

GCATCATAAAATCCAACGCTCTTAGTTACTTACCTACCCCTGTTTCCAGATTCCCGAGGG

AGCGCCGCGCAAGAAAAAG

>FBtr0079731 type=five_prime_untranslated_region; loc=2L:870**5572**..8705694; name=CG9314-RA; MD5=56ba8bbcf31e7ee081dd17223f58d1a4; length=123; parent=FBgn0032061; release=r5.57; species=Dmel;

tgtctcttgccgacgggaccaccttatgttatttcatcatgCACAATTGACAAGCAAAATCACTCACAAC

GCTTTTGTTAGCAGTTGAAATCAACTGTGTGAAATATTTTTTTTGAAAATAAATTTTTTT

TTAGAAATTTTAAGACCAAAGACAAAATTGAAAA

>FBtr0079773 type=five_prime_untranslated_region; loc=2L:916**4235**..9164347; name=CG13108-RA; MD5=6e8085be8e1643e5368e3961910cd699; length=113; parent=FBgn0032100; release=r5.57; species=Dmel;

tgtctcttgccgacgggaccaccttatgttatttcatcatgCAGCTTTGAGACGCAGCAGCCACAGCAGC

AGCAAAAGCAGCAGCAGCAGCCGCTGCAGGATTTAGCGGTCGGTTCAACCAGGAGCAGCA

GGAGTAGAGAATTTTCGCGTCAGA

>FBtr0079879 type=five_prime_untranslated_region; loc=2L:992**1633**..9921740; name=CG4592-RA; MD5=adff1176ce9d6260a89693d9e0262c70; length=108; parent=FBgn0032162; release=r5.57; species=Dmel;

tgtctcttgccgacgggaccaccttatgttatttcatcatgATTTGGTTTGCAACTCGACTTAATATATG

ATAAACAGGTTAATTTGTTTAAATTCCTGCCCGTCGCTCAGTGCGTGATTGCTGCTCCAG

ATCCAAAGTCGTCCAAGTC

>FBtr0079969 type=five_prime_untranslated_region; loc=2L:complement(10092236..1009**2389**); name=CG13133-RA; MD5=f2934b060c5fa326b120ddaf2abe01ba; length=154; parent=FBgn0032181; release=r5.57; species=Dmel;

tgtctcttgccgacgggaccaccttatgttatttcatcatgGCGATTACCATAACCTCATCCACTCAGTT

AGCTTGTAAGAGTCGAACCGGGCGGAGGGCAACGGTTTTTTTGGCCACATAAGCCAAAAA

TCAACACGCTTGCTCCGTGTGTCCAACGAACCGGAGTCCGTGCATCGGGAACCGGTGGCG

CAGTC

>FBtr0079987 type=five_prime_untranslated_region; loc=2L:1030**6922**..10307044; name=CG4968-RA; MD5=15bcf94429caf16d4c0bfba1807db551; length=123; parent=FBgn0032214; release=r5.57; species=Dmel;

tgtctcttgccgacgggaccaccttatgttatttcatcatgGACATTTGATGCGCCTACACATACATATG

ATGAACAGGAATTCTTTTTAGTTCATCAAATGAATACAAAGTAAAGTAAATTGAAAGGAA

GCGCCGATCGCAGTTGTTCAGTATTCAGAGAAAC

>FBtr0079992 type=five_prime_untranslated_region; loc=2L:1033**1471**..10331609; name=CG5037-RA; MD5=c4a6d8a21491faccbfb593c57a848795; length=139; parent=FBgn0032222; release=r5.57; species=Dmel;

tgtctcttgccgacgggaccaccttatgttatttcatcatgTTTTTCTCATTTTGAGATATGGTAACACT

TAAGCACAATGTGATTTAAAAAACCATAACATTGTATATAAAAGTAATAGAAATCCAAAC

ACAACTAGAAAAATGAAATAACCGTAGACCTAACTAATAAGTGAATAATA

>FBtr0337004 type=five_prime_untranslated_region; loc=2L:1050**7081**..10507193; name=CG6094-RB; MD5=08902618ffd6b01a70129035505f1ae8; length=113; parent=FBgn0032261; release=r5.57; species=Dmel;

tgtctcttgccgacgggaccaccttatgttatttcatcatgCTAAGTTCGGTCACACTGCACGCAACTCA

CCTCGAACAGCTGTTCTGTGGTGAACACAAACGTCGTTCTCGTAACATTTAATTTAGCTT

AACTCATTGAAATTTTGGCATAAA

>FBtr0080112 type=five_prime_untranslated_region; loc=2L:1073**2653**..10732817; name=Dpy-30L1-RA; MD5=25be7dcc4cddf6007513f4a7f27c7eee; length=165; parent=FBgn0032293; release=r5.57; species=Dmel;

tgtctcttgccgacgggaccaccttatgttatttcatcatgACAGGAATTCGATGTATAAGTAAATTTTC

GTGTCATCGATTCCCGACATGAATGCGACAACTCTGCAGACCGAGAATGTAAATTTTGAT

CGAATCGAATTTGTTGGACTGCTGCAAAGACGCCAAGTGAAAACCGAGATTTGCTGAACA

AAAGGAACACATTGCC

>FBtr0340557 type=five_prime_untranslated_region; loc=2L:complement(10910536..109**10648**); name=CG14069-RB; MD5=17f34385bf0f5622ba1239b18e41d985; length=113; parent=FBgn0032315; release=r5.57; species=Dmel;

tgtctcttgccgacgggaccaccttatgttatttcatcatgTTTTCAGCTGCTTTTAAAGTTTCAATGCA

AATCAAGAACAGCTGTTGGAACTTAAATGAACAGCTAGTAGCTTAGATGGCGCTTATTAG

CTCAGTTGCCTGCTATTTGTCGCC

>FBtr0080200 type=five_prime_untranslated_region; loc=2L:1113**0737**..11130864; name=CG4751-RA; MD5=52328f450af8fbee846a4c44d2fd9392; length=128; parent=FBgn0032348; release=r5.57; species=Dmel;

tgtctcttgccgacgggaccaccttatgttatttcatcatgAATGAGTTTCGGTATTCTGGGTAGAAAAA

GCAGCTACCTTACTGCATCCATCAGTTTTTTTTCGCAAAACTTAAGTATCTTGGAAGTAA

AATATCCGTAGAATTGCACAGAATTCGCGACAAGAACGC

>FBtr0343614 type=five_prime_untranslated_region; loc=2L:1117**9032**..11179231; name=CG4788-RB; MD5=dad7c87675ad285aca78c0df80be1282; length=200; parent=FBgn0032354; release=r5.57; species=Dmel;

tgtctcttgccgacgggaccaccttatgttatttcatcatgTGATTCCCTAACGCGTGGTTCACACTACA

AAAAAAAAAGACAACGCTGAGTTTTTAACATTAAAAACGTTTTTTAAACCACTAAAACGA

GAAAAATAAATAAAAAATAAGTTTGTGCGTGATCGAAAAGCGTTGGCCGATTTGCAGAGG

AGCAGGCAATTCACAATCAGCTGCCGTGAGAACTGTCGTATAGCCAAGACA

>FBtr0333093 type=five_prime_untranslated_region; loc=2L:complement(12427568..1242**7709**); name=CG5446-RB; MD5=c821c12b5da1f67e38e09a0a8fce4f00; length=142; parent=FBgn0032429; release=r5.57; species=Dmel;

tgtctcttgccgacgggaccaccttatgttatttcatcatgCTATCGAACCAAAGTCCGCCCCTATGCTT

GATAAAGAAATTAATTTGCAGAAGTGAAACAGCCGCAGCCGCAAACAGAAGCAAAAGAGC

TAGAAGTGATAGCATTTTTGTACTTTTATACAGTGCCACAAAGAAGCCACAGA

>FBtr0334114 type=five_prime_untranslated_region; loc=3L:2207**5820**..22075932; name=msopa-RB; MD5=ba1b896c2fac1679c874bc3442386192; length=113; parent=FBgn0004414; release=r5.57; species=Dmel;

tgtctcttgccgacgggaccaccttatgttatttcatcatgAGATCATCGCCAGACATACGATTTGCATT

GGTTCCACCTATTGGCTCCACCGGATCAGTGAGAGCTATAAAGGGCCCCACCGCTTTCGC

CCTGGCCTTCATACCATACTCGCC

>FBtr0072630 type=five_prime_untranslated_region; loc=3L:121**2827**..1212951; name=LysE-RA; MD5=ace5111369f419024a476cb378ddc706; length=125; parent=FBgn0004428; release=r5.57; species=Dmel;

tgtctcttgccgacgggaccaccttatgttatttcatcatgAATATGGTGATCACCTTATCACGGTTGAG

TACTTATACGTTCTTCTTAATAAGGTGATGGTTCAGGGTATATAAGGGCCCATTGCGAGG

ACCTCTCCATCAGTTTACTGTGGTATTCAAATCAAA

>FBtr0078849 type=five_prime_untranslated_region; loc=3R:complement(778219..77**8404**); name=UbcD6-RA; MD5=1afc632f91d19279ee478f349a07ddd0; length=186; parent=FBgn0004436; release=r5.57; species=Dmel;

tgtctcttgccgacgggaccaccttatgttatttcatcatgCTCTACGTGATAGTATCGGGGAAACATCG

GTTTTCGTTCCAGCTCTATACCACTGCTGCCCGAGTTTGCCCTCAATTAAAAATAAATTA

CAAAATTTCATCGTTACCGTTCGCTAAACGCAACGCATTGCCCAGGCGTCCGAGTTCCAA

ATCCAACACAACACGAGTGGTAGTATCGCTGTGAAAA

>FBtr0077828 type=five_prime_untranslated_region; loc=2L:21**30813**..2131011; name=GlyP-RA; MD5=feff8acbe540a578ad405878620e4fe1; length=199; parent=FBgn0004507; release=r5.57; species=Dmel;

tgtctcttgccgacgggaccaccttatgttatttcatcatgAAGTGAGAATCGCTCCGGATTTTGCTGCG

AACGGTCGTCTGTTCAGGTTCAGGTCTGGGTTGTGTCTATAGTCTCCGTCTCCTCTTCGC

TGTCGCACACGCAAACGGGTCTACGCCACACACACACGCGCACCCACGCAACGGAATCAG

AGAACTATATAGAGCCGCCACAGAGAAGGGACATACATAACTAGGACACC

>FBtr0330167 type=five_prime_untranslated_region; loc=3R:complement(22738483..227**38608**); name=Ets97D-RB; MD5=ca3788fe976f5960c7b4afa4b8365d7d; length=126; parent=FBgn0004510; release=r5.57; species=Dmel;

tgtctcttgccgacgggaccaccttatgttatttcatcatgAGTATTATTCGGTATTGCCACACCCGACG

TACACGGTCACACTGAAGATGCAACAATAAATCCACGAGACAAATTTGTTAAAAACATTT

TGTTAATATAGGCAGTTATTTGCGATTTTAAGCCGCG

>FBtr0073263 type=five_prime_untranslated_region; loc=3L:complement(4138815..413**8996**); name=Rop-RA; MD5=7aa010f5f3715536cd320e9ada364601; length=182; parent=FBgn0004574; release=r5.57; species=Dmel;

tgtctcttgccgacgggaccaccttatgttatttcatcatgACTAAGGCTAAGTCATCAAACGAAAAAAA

TACAGAAAAATTGGCCAAGGAATAGATAAAATTCTTTAAGTCAAGTTAGCCCACGCGAAA

AAGCGAACGGAATAAGCTAAAAAGACATATTTACACGCAGTGTGTTCATAATCGAGTTGC

CTTTGCGCCCGTGACCAATAAAACGCAGTGAAA

>FBtr0083010 type=five_prime_untranslated_region; loc=3R:complement(10715780..1071**5927**); name=CycC-RA; MD5=40872dfdd2b7a14f096394fcea487f9f; length=148; parent=FBgn0004597; release=r5.57; species=Dmel;

tgtctcttgccgacgggaccaccttatgttatttcatcatgGCCCTGGTGTGCACACACTGGCTGAAACG

GCAACACAATTTTTTCGTTTTTCCTGGTTTTCCGGCTTGCTATTGAAATAAACACCTAAT

TTGGATAGCTAACACGAAAATATTGGCCGACCTAATCAGCTAGGCCTACTAGTTACGAA

>FBtr0070441 type=five_prime_untranslated_region; loc=X:249**8230**..2498335; name=Zw10-RB; MD5=a84281086b8d9dc65ba35f18d697743a; length=106; parent=FBgn0004643; release=r5.57; species=Dmel;

tgtctcttgccgacgggaccaccttatgttatttcatcatgCTATCGCTTTTTTGAACAATCGTTGGTAG

CAACAGGGAATTGCAGAAACCAGTTGTCTTGGGTTTCGCAAGAATTGGAAAAGGAGGATC

TGGTCAAAGAGCAGGAC

>FBtr0344737 type=five_prime_untranslated_region; loc=X:39**3991**..394185; name=svr-RL; MD5=f47562813235d6dc3b9cd2cd9aa7eaf4; length=195; parent=FBgn0004648; release=r5.57; species=Dmel;

tgtctcttgccgacgggaccaccttatgttatttcatcatgTCAAACAGTTGACTTTTCGAGCCTGAGCT

GCAACGACGACCCACGACGAACGGAGGCCCGACGGACATATAAGAAGAGAGCGAGTTGTG

TGCGGGAGAGAGCTGAGAAGAAGCGGAGGAAAAATAACTGTTTCTCGCTCAATTCGCACG

CCAGTGATAAAAACAACATAATCTCGACAAGATTGCAAATCGCAAG

>FBtr0087047 type=five_prime_untranslated_region; loc=2R:1278**6040**..12786149; name=inaC-RA; MD5=bea8eb412d9b71255a506ab2e05662af; length=110; parent=FBgn0004784; release=r5.57; species=Dmel;

tgtctcttgccgacgggaccaccttatgttatttcatcatgAGGGTCTTCACCTGACCGACCGGGTCAGT

TTCTAGACGGGCTTGAAACCGTACGGTCTGAGTCAGCTTTTTCTCTTCACTAGCTATCCA

ATTCCCTGACATTATCTTTTA

>FBtr0081316 type=five_prime_untranslated_region; loc=2L:complement(19795747..1979**5851**); name=fs(2)ltoPP43-RA; MD5=1d8a20a5ad94df4431cf0d734d773486; length=105; parent=FBgn0004811; release=r5.57; species=Dmel;

tgtctcttgccgacgggaccaccttatgttatttcatcatgGCAGATATATTTTGGCATGCCCCGAACGG

TCACACTGCGCGTCGCACACTTTTAATTGTTTCGCTTCGCCTTTCGTTGTTTGTCGTATT

CTCCGAATTAATCAAA

>FBtr0071350 type=five_prime_untranslated_region; loc=X:complement(9000481..900**0637**); name=Bx42-RA; MD5=ec48555006d51a18aef194bb0b9f70e5; length=157; parent=FBgn0004856; release=r5.57; species=Dmel;

tgtctcttgccgacgggaccaccttatgttatttcatcatgTTATTAGCAGTCTGGCAACCCTACACACC

CGTACGCTTGTGTGTGTCCGTGTGCGTGCGTGTTTTTTTTTTCGACTTTTCCATTATAGT

TTTTGTGGCTTGCATATTGAAAAGTGATTTCACATAAGAATTGATTCGCCACAAATAACA

GATAAGCT

>FBtr0084087 type=five_prime_untranslated_region; loc=3R:1721**4602**..17214736; name=bap-RA; MD5=428fb0257948e9bd30d7f2936658ffee; length=135; parent=FBgn0004862; release=r5.57; species=Dmel;

tgtctcttgccgacgggaccaccttatgttatttcatcatgTTGAAGCACATTCGCTGCGCAGCCCTCGA

GTCGTGAAGATCGGAAAATCAATAGAAATCCGTAGTGAACAAGTGTCGGAAAGGCCAAAG

ACAATACCTATATATATATATCCGAAATATTCAGAAAATTCGAAAA

>FBtr0079862 type=five_prime_untranslated_region; loc=2L:complement(9494616..949**4796**); name=Gdi-RA; MD5=73bcfbc357db5e9a910cd09973a4cc8b; length=181; parent=FBgn0004868; release=r5.57; species=Dmel;

tgtctcttgccgacgggaccaccttatgttatttcatcatgGCGTGACGGCAAACGGGCAGCGCAAAGCG

GACGAGCGAAAGGCGAACGAGTAGAGAAAAATTATTGAGCGCAGAGAAAAGTCCACCAAA

TCGAACGTCGCAGCAAGTGAAAAGGGCGAAAACCGGATAAAAAAAATATATAAATTACTG

TGATTGCAATATATAAATAAGTGCGATCCAAT

>FBtr0078725 type=five_prime_untranslated_region; loc=3R:complement(1542335..154**2527**); name=cas-RA; MD5=09a993f2a318f1fa2bd8ebfbfb5fe218; length=193; parent=FBgn0004878; release=r5.57; species=Dmel;

tgtctcttgccgacgggaccaccttatgttatttcatcatgGAAGCAGTTTGAGCCTCGCAGCCGAACTT

TGAGGATCGCTGAGACGAGACGCCGTGCAAGAAAGTCGATCCAAAATCCGAAATTCGAAA

TCCGTGCTGTGATCTGTGCTGAATATAGTGAAGAAATCTTAAAGAATTTTCAAAAAGAAA

TCCCAGTGCTGTGCTGAATATATCCGCGAAAGTGTGCCACAAAA

>FBtr0100339 type=five_prime_untranslated_region; loc=3R:complement(22701335..2270**1464**); name=Rb97D-RH; MD5=9cd372edb4e24d0dcb6ebb11b4bc2360; length=130; parent=FBgn0004903; release=r5.57; species=Dmel;

tgtctcttgccgacgggaccaccttatgttatttcatcatgGGTGTATCGAATCAATGATTCGCTTCCTG

ACAGCACAATTTTCACTTCGCTTCAACCGTTCGACGTGCAAACTGGTCGTTTTCGTTTCT

TAATTTCGGTTAAATTTAGTGATAATTAATTTGCTAACACA

>FBtr0074819 type=five_prime_untranslated_region; loc=3L:2012**2281**..20122408; name=gig-RA; MD5=5e4b65c3d963147920538d2b7d9cb6ef; length=128; parent=FBgn0005198; release=r5.57; species=Dmel;

tgtctcttgccgacgggaccaccttatgttatttcatcatgCAGCTGTTTAGTGACTTCCTCTGTTCTGT

GCTGTGTATAAATTGTTTTTAGGACCTAGGCTAGGAATTACTGGTTGCACACACTCAGCG

CCACAGCTCCACCGACCACCGCCCTTGCAAGGACCCACC

>FBtr0087769 type=five_prime_untranslated_region; loc=2R:complement(9092714..909**2842**); name=lat-RB; MD5=4200c62cf982e6a0cba9370e095756d8; length=129; parent=FBgn0005654; release=r5.57; species=Dmel;

tgtctcttgccgacgggaccaccttatgttatttcatcatgATTTCATTTCGCGTTCCTACCGGTGAAGA

AACTTTAATTGGCACAGAGCTGACCTAAACAAATAAATAAATAAACGCAACCCGGCGACT

TAAAGACTCCACGACATCGGGCCGCAGTGCAAAGCCAGCG

>FBtr0086307 type=five_prime_untranslated_region; loc=2R:complement(16150227..1615**0388**); name=PCNA-RA; MD5=7c7b22a981522fdffcd057b0fdeccd5e; length=162; parent=FBgn0005655; release=r5.57; species=Dmel;

tgtctcttgccgacgggaccaccttatgttatttcatcatgGTGGCTTTTCACATCCCTATCCCGCTCAT

TTAGCCCGCCTGAAAGTAAAAAAAAAAAACAGCCCACGTTAATCATTCATCCCAAAGTCA

CAGCCGCGGTAACATTACTGCTGTTAAATTCTTAAGCCCGTCATCAGTATTTAAATAATA

AAACACATTCAAT

>FBtr0082671 type=five_prime_untranslated_region; loc=3R:complement(8453244..845**3423**); name=Vha55-RA; MD5=1a91a25757127846c30cc7d8a0ef2082; length=180; parent=FBgn0005671; release=r5.57; species=Dmel;

tgtctcttgccgacgggaccaccttatgttatttcatcatgCGACTTCGCAGGCCAAAATTTTGCGTGTG

TGTCGCTCAGTACTGTTTCGTAGCTGAAAAATTGTATGTGCTTGTAAAAGACCACGTCCG

AATAGTGGCTAAAAGCTAAAATCGTATCTAATTGCAGCTCGAAATTTGCAGTGCACTTTC

AAGGTAGAGGCGCAGAAAAAGTCTATCCAAG

>FBtr0084902 type=five_prime_untranslated_region; loc=3R:2163**6818**..21636926; name=CG5024-RA; MD5=027b3709c50ef94df01c525cea927d19; length=109; parent=FBgn0039373; release=r5.57; species=Dmel;

tgtctcttgccgacgggaccaccttatgttatttcatcatgGTCAATGAAAAGACAATATTTAAACACAA

CTATTTTCGAAAAACTAAATTTTAAAATTCATCTTTTCGGGAAATCCAAATTTTAGCCCA

AGGTATACTTACAATTTGCC

>FBtr0084984 type=five_prime_untranslated_region; loc=3R:complement(21748220..2174**8356**); name=CG14546-RA; MD5=5eb0bb779f12d4e0bf779621ea801b8a; length=137; parent=FBgn0039395; release=r5.57; species=Dmel;

tgtctcttgccgacgggaccaccttatgttatttcatcatgAAATATCTGAAAAAAAACAAGCTATTTAA

TTTCAAAAGAGCTTACATCCACATGATGCGAAGGCAAAGATTTCATTACAATAACATGAG

CCGCCAATTTTAAATCGATCTACATCCTAAAAATTCGTCAAAAAAGTC

>FBtr0085055 type=five_prime_untranslated_region; loc=3R:complement(22451243..2245**1374**); name=TwdlL-RB; MD5=83e75b59e2c654bbe82aa76078200cfe; length=132; parent=FBgn0039437; release=r5.57; species=Dmel;

tgtctcttgccgacgggaccaccttatgttatttcatcatgAGAAGCAATTTGTGCTCCGAACCAAGAGA

ACCTCAAACCCAAAACTAAAGATGCGCGCCTTCATCGTAAGTGGTGTAGCCAGGACTTAA

GACGAATTCACAACTAACGGATAACCCTTGAATCCTGTAGGTA

>FBtr0085043 type=five_prime_untranslated_region; loc=3R:2256**3332**..22563530; name=CG14247-RA; MD5=cfaf69007da89e1a044b5ddd64d55b62; length=199; parent=FBgn0039454; release=r5.57; species=Dmel;

tgtctcttgccgacgggaccaccttatgttatttcatcatgAATCAGTCGACTCGGGTTTTTCAGCAGGA

AGCACACACATAAATTTATTAACGAACCCAGCTTATGGCCAAATGATGAGAAGCGAGTGA

AAAAATATCTGAATTTATCACCCAACTGCAGTTCGTAAAAAAAAAAAAAAAAACAAAGAA

AAGAAAAGTAAAATCAGAGCGAACGCGAACTTTGTGTGTCATAATAATAA

>FBtr0085728 type=five_prime_untranslated_region; loc=3R:complement(26521104..2652**1220**); name=CG12069-RA; MD5=f9f853c8bde76c7f654d3dbee82928ea; length=117; parent=FBgn0039796; release=r5.57; species=Dmel;

tgtctcttgccgacgggaccaccttatgttatttcatcatgGGAGCGTCAGGTCATTTTCAATCCGAGCT

TTCGTAATGCTTTAGACAAAAATTCGAAATTCTGGTATAGTTAATTGACGCGATCACTCA

GAACTCTAATACGCGAGTTGTTGTCGCA

>FBtr0332202 type=five_prime_untranslated_region; loc=2L:complement(11268847..1126**9002**); name=cmet-RC; MD5=25d88d93c76cfec36f9519163376c314; length=156; parent=FBgn0040232; release=r5.57; species=Dmel;

tgtctcttgccgacgggaccaccttatgttatttcatcatgTATACATCCCTGATTTTGCAAAGCGGCAT

TTGTTCGAATTTAACGAGCAACAAAGATGTCGATCAATGTTTGTTTACTGTAAAACATTG

AATTTTATGTTTGTTTATGAACTGCGTTCTGTTTATCATAAAGCATTGAGACCAAGAAGG

CGCCAAG

>FBtr0071324 type=five_prime_untranslated_region; loc=X:complement(9213156..921**3256**); name=c12.1-RA; MD5=6cc63fff0f3dc9594e4dda43ca3d413f; length=101; parent=FBgn0040235; release=r5.57; species=Dmel;

tgtctcttgccgacgggaccaccttatgttatttcatcatgGATTATTGGTACTGTTGATACATCAAACA

AAGCACGCAGTTCGGAAATTCATTTCACTTTTCATCAATTTAACAATTAAACGCATAACT

AGCTAGTTGAAA

>FBtr0332591 type=five_prime_untranslated_region; loc=X:complement(15343801..1534**3916**); name=Scamp-RC; MD5=13c58f47d3a28e34da8f8143a94e479b; length=116; parent=FBgn0040285; release=r5.57; species=Dmel;

tgtctcttgccgacgggaccaccttatgttatttcatcatgAAAACCGACATCGATAACTGAGCAGAGTC

TTAGGGAAAATATACACAGCGAAAAGCAAAGTGCGAGTCAGTGCGTTTTTCTGGTCATTG

AACAGCCGTATCCAAAGTAGAATTCCC

>FBtr0073763 type=five_prime_untranslated_region; loc=X:1322**3857**..13223997; name=Jafrac1-RB; MD5=d7a95be62ec72417351fea1694b28dff; length=141; parent=FBgn0040309; release=r5.57; species=Dmel;

tgtctcttgccgacgggaccaccttatgttatttcatcatgAATCCATTCGCGAATTTCAACTCGTGCCG

AAAGGTTTGTTTGTGCGAAAGCTGAAGCTGCGAGAGAACGAACAAGAGGAAGCCCACGCG

GAAGTCAAGCAATTACCGAGAATTACTAAGAGAAAAAATATAACTGTTTAAA

>FBtr0331301 type=five_prime_untranslated_region; loc=X:complement(824018..82**4131**); name=CG3708-RB; MD5=f30a957913b2dfa97f4e72b2913c8785; length=114; parent=FBgn0040345; release=r5.57; species=Dmel;

tgtctcttgccgacgggaccaccttatgttatttcatcatgAGCTTTGATCTAGCATAGTAGTTTCCAAG

TGCCCAGTCGCAGAGCTGGAATATACTTGTAGATTTATATACATATATGTTGCCAAATTT

CGAAGTTATTGCACCGAATTGCACA

>FBtr0080198 type=five_prime_untranslated_region; loc=2L:1111**4014**..11114182; name=Gr32a-RA; MD5=bad206762033666cba3ae62474108f30; length=169; parent=FBgn0041246; release=r5.57; species=Dmel;

tgtctcttgccgacgggaccaccttatgttatttcatcatgTCTCCATTGGCAATATTCAATAATTCACA

ATGCCAAGCAATTACTCTAATGGCAAAGTCTCGGGCCCAAGTGGGCCCACGACAAAATCA

GATAGCTCAGAACCCACTTAAATGAACGCTGTTCAATCCAGTTAGTGCAATCCTTTCGTC

CTGTCAGTGCCACGTTGGCC

>FBtr0085410 type=five_prime_untranslated_region; loc=3R:complement(24977412..2497**7516**); name=ligatin-RA; MD5=b810689ce0aacc9bfcf53ec11903ec99; length=105; parent=FBgn0041588; release=r5.57; species=Dmel;

tgtctcttgccgacgggaccaccttatgttatttcatcatgAAATAGCTAAAGCAGCACCCCCGTTTGCC

AGTTGTTTGTTTTCACTGTGCATGTGCCACTCCAAACCATTTCACGAACTCCTCAAGTGC

ACACCACCAAACACAG

>FBtr0331978 type=five_prime_untranslated_region; loc=2R:complement(14859939..1486**0042**); name=CG15107-RB; MD5=7569e15ce7e9d824dc7066bb1cfeaffe; length=104; parent=FBgn0041702; release=r5.57; species=Dmel;

tgtctcttgccgacgggaccaccttatgttatttcatcatgCTTAATTGTTTGTGTTATCGATAGCTCGT

TGCTTTATTGCCTTTTTGTGGCGTCTGTTTCAATTTGTTTGTTTTCAATTGTTGGTCATA

ATTTGGATCCCCAAA

>FBtr0086107 type=five_prime_untranslated_region; loc=2R:252**4017**..2524193; name=Bap170-RA; MD5=2e501aff7ca96df8c27cebbecab62f5a; length=177; parent=FBgn0042085; release=r5.57; species=Dmel;

tgtctcttgccgacgggaccaccttatgttatttcatcatgCGGTCCAATTGCTGACAGCCCTGCTCGTG

AATGCAACTCTGCTGTAGCGCGTCAGCGTCGCGTTGTAATTTTTGTTGAGTTTTTTAAAT

AATTTTAATTAGCATCTGCGTGTCTTACTATTAGAATATCATGTGTTAAGTGTTTCCCAT

TCACGCCACCACACAAAAAGATCCAATC

>FBtr0082417 type=five_prime_untranslated_region; loc=3R:745**2611**..7452715; name=CG18764-RA; MD5=9ed3e70db53c739c46ee57fec85153a3; length=105; parent=FBgn0042205; release=r5.57; species=Dmel;

tgtctcttgccgacgggaccaccttatgttatttcatcatgATATACATACATATGTGTAGTGGATGCGT

TTACAAAACAATTGGTTGTTTATATTCGAATTAAACTGAAGTTGGGCTAAACTTCGGTTG

ATTTTCAACTAACATT

>FBtr0273268 type=five_prime_untranslated_region; loc=3R:1638**4020**..16384119; name=CG5180-RB; MD5=f909fa8622aaa668f2f544ce2f966bdf; length=100; parent=FBgn0043457; release=r5.57; species=Dmel;

tgtctcttgccgacgggaccaccttatgttatttcatcatgTTGTGGCGCAGCCAACTTCACGCGAGACG

CAGCGAAAACAAAAAATACTAAGCTGAAGTTAATTGAGAAAACCCCAAATAAAACGCGAG

TGAAAGGGACC

>FBtr0080391 type=five_prime_untranslated_region; loc=2L:complement(12421705..1242**1862**); name=vir-1-RC; MD5=d9031b177be92920322e80d556f85bb9; length=158; parent=FBgn0043841; release=r5.57; species=Dmel;

tgtctcttgccgacgggaccaccttatgttatttcatcatgGCGCATTTGACGATTGTTTGTCTTTCCGC

ACAGAACAAAACTGAACAGAATCGAATCCGGTTGCCGGTCCACATCAACGCATCAAAGCT

TTCAGCCCTAAATAAACTATACAAATTAAACAAATTATAAGCTTGTGAAAAAAAAAAAAC

CAAACAAAA
