# Supplementary material for: Genome ARTIST: a robust, high-accuracy aligner tool for mapping transposon insertions and self-insertions
Source: Mob DNA. 2016 Feb 5;7:3. doi: 10.1186/s13100-016-0061-0 (PMC4744444; doi:10.1186/s13100-016-0061-0)
Supplement: Additional file 4: — Manual annotation of reference sequences. The file summarizes a concise approach useful when loading and annotating genomic and/or transposon reference sequences in a Genome ARTIST-friendly format. (DOC 27 kb) [file 13100_2016_61_MOESM4_ESM.doc]

**Additional file 4**

By default, for each individual genome or transposon file which is loaded in its databases, Genome ARTIST computes a *.raw* file containing the nucleotide sequence and a corresponding *.gene* file for the specific annotations of the sequence. These files are accessible by following either the path *resource > raw* or *resource > gene*. The annotations may refer to genes or to any other nucleotide structure of the loaded sequence. Information about several features such as the item's name and ID, the start and the last nucleotide coordinates on the reference strand, its orientation relative to the reference strand and the cytogenetic localization are offered. Actually, the *.gene* file contains a list of structured annotations, where every single annotation is, in fact, a linear arrangement of features. The user may annotate virtually any reference nucleotide sequence in order to harness the graphics of Genome ARTIST. The *.gene* file corresponding to the *.raw* file of interest can be modified or completed by the user by simply changing a particular feature of a single or of multiple annotations from the list, or by eliminating or adding a whole annotation row.

As an example, we present herein how to annotate the *P{lacW}* transposon using data available in FlyBase under the *FBtp0000204* ID. In the *Segments and Size* section, a list of seven DNA segments forming *P{lacW}* is provided. When individually accessed, each corresponding report displays the nucleotide sequence and the extent (coordinates) of the respective sequence inside *P{lacW}*. Using these data, we compiled an annotation *P{lacW}.gene* file which contains the main modules of *P{lacW}*:

5'TR-P5' ; none ; 0 ; 581 ; F

lacZY ; none ; 593 ; 4015 ; F

Hsp70Bb ; none ; 4025 ; 4256 ; F

mini-white ; none ; 4281 ; 8407 ; F

Ori/Amp ; none ; 8432 ; 10270 ; R

3'TR-P6.1_int.del. ; none ; 10309 ; 10468 ; F

3'TR-P3' ; none ; 10469 ; 10690 ; F

none ; none ; 10691 ; 10691 ; none

The annotation line referring to the first nucleotide of the sequence must start with the coordinate 0. Also, the last annotation line is read and graphically showed by Genome ARTIST only if it is followed by a gratuitous annotation line (the underlined annotation), which does not contain any particular feature. The last nucleotide of *P{lacW}* is 10691, but it becomes 10690 when starting from 0, therefore the value 10691 in line 8 is written just to over-pass the last annotated nucleotide.

Any modifications regarding the number of annotation lines of a certain genomic or transposon .*gene file* (by adding or deleting specific annotations) must be followed by an accordingly adjustment of the number value standing for the *[GENE NUMBER]* feature in the dedicated genome or transposon file descriptor from the *paramsIndexFisiere.txt* file (8 is the respective value for the *P{lacW}*, since there are 8 rows of annotations). The file *paramsIndexFisiere.txt* is available in the installation folder of Genome ARTIST.
