# Supplementary material for: Genome ARTIST: a robust, high-accuracy aligner tool for mapping transposon insertions and self-insertions
Source: Mob DNA. 2016 Feb 5;7:3. doi: 10.1186/s13100-016-0061-0 (PMC4744444; doi:10.1186/s13100-016-0061-0)
Supplement: Additional file 5: — Screenshots of Genome ARTIST and iMapper mapping graphics. The file contains screenshots from Genome ARTIST and iMapper graphical outputs (A and, respectively, B and C) when both tools are fed with query sequences containing a simulated one nucleotide deletion. (PDF 322 kb) [file 13100_2016_61_MOESM5_ESM.pdf]

Additional file 5

A

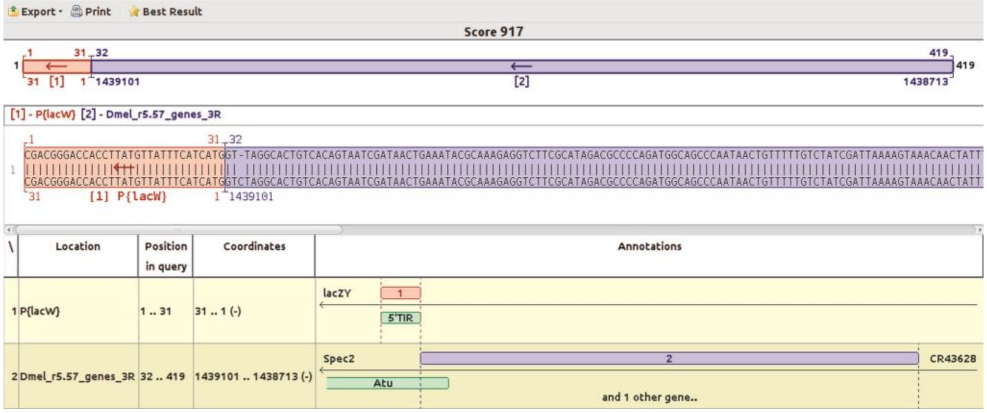

B

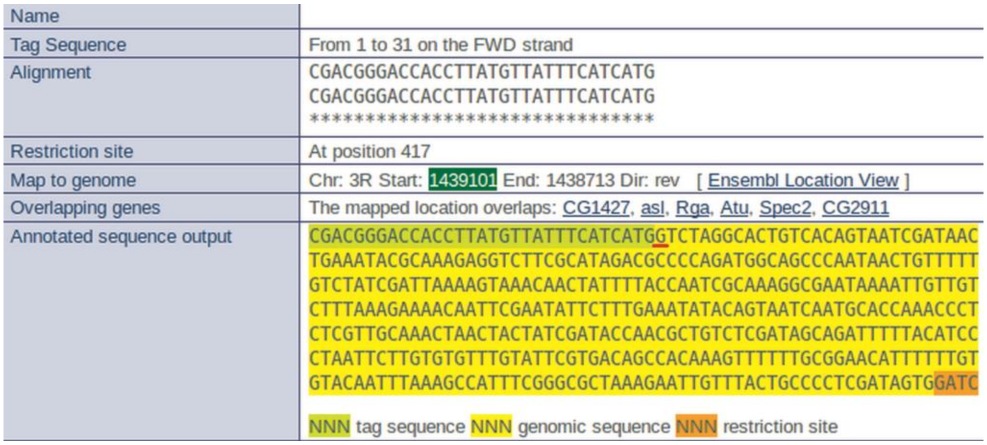

Name	
Tag Sequence	From 1 to 31 on the FWD strand
Alignment	CGACGGGACCACCTTATGTTATTTCATCATG<br>CGACGGGACCACCTTATGTTATTTCATCATG<br>*******************************
Restriction site	At position 417
Map to genome	Chr: 3R Start: 1439101 End: 1438713 Dir: rev [Ensembl Location View]
Overlapping genes	The mapped location overlaps: CG1427, asl, Rga, Atu, Spec2, CG2911
Annotated sequence output	CGACGGGACCACCTTATGTTATTTCATCATGGTCTAGGCACTGTCACAGTAATCGATAAC<br>TGAAATACGCAAAGAGGTCTTCGCATAGACGCCCCAGATGGCAGCCCAATAACTGTTTTT<br>GTCTATCGATTAAAAGTAAACAACTATTTTACCAATCGCAAAGGCGAATAAAATTGTTGT<br>CTTTAAAGAAAACAATTCGAATATTCTTTGAAATATACAGTAATCAATGCACCAAACCCT<br>CTCGTTGCAAACTAACTACTATCGATACCAACGCTGTCTCGATAGCAGATTTTTACATCC<br>CTAATTCTTGTGTGTTTGTATTCGTGACAGCCACAAAGTTTTTTGCGGAACATTTTTTGT<br>GTACAATTTAAAGCCATTTCGGGCGCTAAAGAATTGTTTACTGCCCCTCGATAGTGGATC<br><br>NNN tag sequence NNN genomic sequence NNN restriction site

C

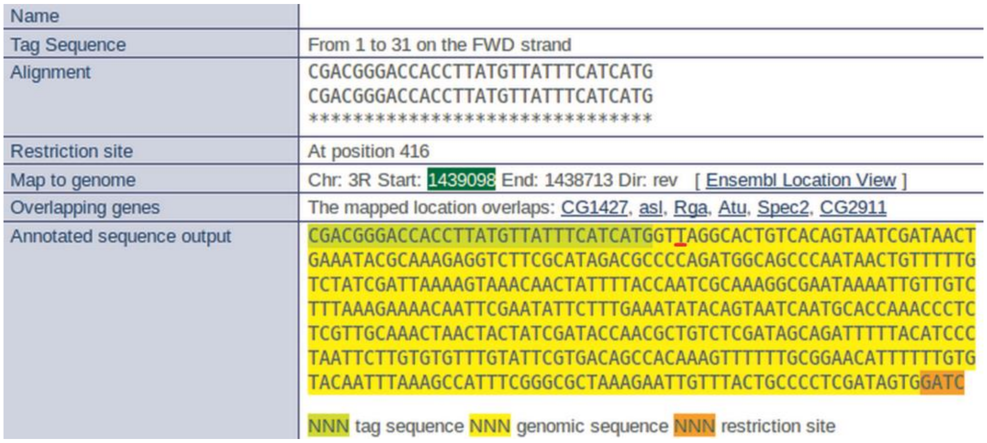

Name	
Tag Sequence	From 1 to 31 on the FWD strand
Alignment	CGACGGGACCACCTTATGTTATTTCATCATG<br>CGACGGGACCACCTTATGTTATTTCATCATG<br>*******************************
Restriction site	At position 416
Map to genome	Chr: 3R Start: 1439098 End: 1438713 Dir: rev [Ensembl Location View]
Overlapping genes	The mapped location overlaps: CG1427, asl, Rga, Atu, Spec2, CG2911
Annotated sequence output	CGACGGGACCACCTTATGTTATTTCATCATGGTTAGGCACTGTCACAGTAATCGATAACT<br>GAAATACGCAAAGAGGTCTTCGCATAGACGCCCCAGATGGCAGCCCAATAACTGTTTTTG<br>TCTATCGATTAAAAGTAAACAACTATTTTACCAATCGCAAAGGCGAATAAAATTGTTGTC<br>TTTAAAGAAAACAATTCGAATATTCTTTGAAATATACAGTAATCAATGCACCAAACCCTC<br>TCGTTGCAAACTAACTACTATCGATACCAACGCTGTCTCGATAGCAGATTTTTACATCCC<br>TAATTCTTGTGTGTTTGTATTCGTGACAGCCACAAAGTTTTTTGCGGAACATTTTTTGTG<br>TACAATTTAAAGCCATTTCGGGCGCTAAAGAATTGTTTACTGCCCCTCGATAGTGGATC<br><br>NNN tag sequence NNN genomic sequence NNN restriction site

A deletion affecting the genomic nucleotide 3 relative to the TIR was simulated for *a P{lacW}* insertion affecting *Atu* gene from *D. melanogaster* [GenBank:HM210951.1]. While Genome ARTIST still reports the coordinate 1439101 as the insertion site (A), iMapper

erroneously displays the T (underlined in red) at the coordinate 1439098 (highlighted with green) as the mapping coordinate for the simulated query sequence (C). Nevertheless, the respective T nucleotide is not intuitively positioned right next to the terminal nucleotide of the TIR (or the tag sequence, highlighted with light green by iMapper). Instead, a G nucleotide, which actually should stand for the real mapping position at 1439101 (B), is somehow confusingly depicted as the one bordering the TIR. Moreover, the deleted nucleotide is explicitly represented by Genome ARTIST, but it cannot be directly inferred from the graphics of iMapper.
